# Developing a job retention vocational rehabilitation intervention for people with long covid: a person-based approach

**DOI:** 10.1136/bmjopen-2025-109740

**Published:** 2026-05-15

**Authors:** Clement Boutry, Julie Phillips, Carina Knight, Jain Holmes, Priya Patel, Richard Morriss, Roshan das Nair, Eleanor Douglas, Charlotte E Bolton, Boliang Guo, Kate Radford

**Affiliations:** 1 Mental Health and Clinical Neurosciences, University of Nottingham School of Medicine, Nottingham, UK; 2 Centre for Rehabilitation and Ageing Research, University of Nottingham School of Medicine, Nottingham, UK; 3 Royal Free Neurological Rehabilitation Centre, Royal Free London NHS Foundation Trust, London, UK; 4 Institute of Mental Health, Nottinghamshire Healthcare NHS Foundation Trust, Nottingham, UK; 5 Health Division, SINTEF, Trondheim, Norway; 6 Nottingham University Hospitals NHS Trust, Nottingham, UK; 7 Respiratory Medicine, Nottingham University Hospitals NHS Trust, Nottingham, UK; 8 NIHR Nottingham Biomedical Research Centre, Nottingham, UK; 9 Centre for Respiratory Research, Translational Medical Sciences, School of Medicine, University of Nottingham, Nottingham, UK

**Keywords:** Fatigue, COVID-19, Workplace, Psychosocial Intervention, Feasibility Studies, Post-Acute COVID-19 Syndrome

## Abstract

**Abstract:**

**Background:**

Long covid affects a significant proportion of people following SARS-CoV-2 infection and is associated with persistent symptoms such as fatigue, cognitive dysfunction and breathlessness which can negatively impact a person’s ability to return to and remain in work. Although tiered vocational rehabilitation (VR) models have been proposed, these are often generic, lack empirical validation and may not address the complex, fluctuating needs of this population.

**Objectives:**

To co-design a VR intervention (the COVID-19-VR intervention) to support return to work (RTW) for people with long covid (pwLC).

**Setting:**

Primary and secondary care.

**Design:**

Mixed-methods target population-centred, person-based approach in three stages: Stage 1: interviews (n=21) with pwLC to identify issues and challenges faced in working with long covid. Stage 2: three co-design workshops with pwLC and service providers to (a) generate guiding principles, (b) identify key intervention features to address work needs, (c) create a logic model to illustrate how the intervention could work and (d) develop a treatment plan and resources. Stage 3: feasibility and acceptability testing in six cases (three critical care admissions, three primary care referrals).

**Results:**

PwLC described work-related problems relating to: fluctuating symptoms (cognition, fatigue and breathlessness), employer, coworker and family’s understanding of long covid and workplace adjustments. We developed a 6-session, 12-week individually tailored, remotely delivered intervention that included vocational goal setting, RTW planning, fatigue/symptom management, financial advice, and where permitted, education for family/employers, employer engagement and negotiation of a phased RTW. Following feasibility testing, changes included accommodating the long-term nature of long covid, addressing unmet psychological needs, and adding content on adjustment, processing traumatic experience and performance/symptom anxiety, with extended delivery including monitoring, review and case coordination.

**Conclusions:**

PwLC may need specialist help to RTW. Our COVID-19-VR appears feasible and acceptable and warrants further evaluation using a staged approach, prior to any definitive effectiveness trial.

**STRENGTHS AND LIMITATIONS OF THIS STUDY**

- The person-based approach and in-depth exploration of vocational support needs during development ensured that the COVID-19-vocational rehabilitation intervention reflects the lived experiences and needs of people with long covid, improving real-world applicability.
- The sample included people with long covid living in the community and those surviving critical care, reflecting lived experiences of people with severe long covid symptoms employed in public sector roles, including teaching and healthcare, disproportionately impacted by the COVID-19 pandemic.
- The study captures and highlights some of the most challenging contextual factors likely to influence broader clinical implementation.
- Most participants were infected with SARS-CoV-2 before vaccines were available.
- The sample was small, and the majority were women, potentially facing additional pressures such as homeschooling alongside work. They may not represent the broader long covid population.

## Introduction

 In early 2020, COVID-19, a SARS-CoV-2, spread globally and resulted in substantial mortality.^1^ The number of confirmed cases reached over 750 million, with just under 7 million cumulated deaths. For those surviving the infection, symptoms, such as cognitive impairments, loss of smell and/or taste, skin rash and joint pain, persist after recovery^2 3^ sometimes lasting months or years,^4^ particularly for those with more severe symptoms during infection.^5^ Long covid, defined as the ‘continuation or development of new symptoms 3 months after the initial SARS-CoV-2 infection, with these symptoms lasting for at least 2 months with no other explanation’^6^ is now a recognised condition, affecting up to 60% of COVID-19 cases,^7^ with 41% having persisting symptoms after 1 year, and 19% after 2 years.^8 9^ It varies in intensity depending on age, gender, severity of the COVID-19 infection and sociodemographic characteristics.^5 10^ It is especially prevalent in public-facing professions such as education and healthcare.^11^ People with long covid (PwLC) report difficulties in their ability to undertake daily activities,^12^ including work, with a Swedish study showing that 9% of COVID-19 cases were on sick leave 4 months post-infection.^13^ In a systematic review and meta-analysis of studies examining activity limitations and participation restrictions within 6 months of hospitalisation following COVID-19 infection, Smith *et al*^14^ identified a 16% reduction in 6 min walk test distance (compared with age and gender-matched norms) and 59% return to employment rate.

The economic impact of long covid is considerable. Since the start of the COVID-19 pandemic, the number of people economically inactive because of long-term sickness has risen to over 2.5 million people, an increase of over 400 000,^15^ with those reporting five or more health conditions (up from 34% in 2019), suggesting many have interlinked and complex health issues, possibly compounded by the COVID-19 infection.

Symptoms such as fatigue, brain fog and breathlessness impact on return to pre-COVID-19 employment.^16^,^18^ *S*ome report reductions in the number of hours or days worked or changes in duties.^19 20^ To support with these difficulties, clinical guidelines were developed by the National Institute of Clinical Excellence,^21^,^24^ and studies developed rehabilitation models for those with long covid.^25^ However, most are based on generic principles, including employer/employee communication, identifying obstacles and solutions, psychoeducation and self-help, functional assessment and prolonged phased return to work (RTW) rather than empirical research, and there is no research evaluating their effectiveness. In one cohort study, only half of those completing the programme successfully returned to work,^26^ with little mention of factors affecting RTW success.

Similar tiered models of care were developed by healthcare providers. For instance, the National Health Service (NHS) England, NHS Improvement (London) and Health Education England developed a 3-tier vocational rehabilitation (VR) model offering guidance for clinicians for supporting RTW post-COVID-19. The tiers represent increasingly complex work-related needs, allowing management to be stratified. At the lower tier, are people whose symptoms are improving, needing only advice and support with RTW plans. This could be provided by occupational health (OH) services or primary care. At the next tier, people whose symptoms persist more than 12 weeks who are struggling to maintain or RTW and have no or limited access to OH. At the top, are people with specialist VR support needs, symptoms persisting beyond 6 months, and those on sick leave, no longer employed or unable to plan their own RTW. A limitation of these models is that they were developed ad hoc. As such, they make assumptions about needs to be addressed at different levels, and what is required from a rehabilitation perspective, without in-depth understanding of the challenges COVID-19 poses to occupational and functional recovery or the implications for clinical implementation.

Given the uncertainty and heterogeneity of long covid presentations, and its impact on work,^14^ there is a need for a theoretically informed, bottom-up approach to intervention development, that is explicitly designed around the vocational needs of pwLC.

We have previously developed and evaluated VR interventions for people with traumatic brain injury (TBI),^27^ stroke,^28^ multiple sclerosis (MS)^29^ and trauma rehabilitation,^30^ and adapted them for remote delivery.^31^ While pre-pandemic VR was typically delivered face-to-face, COVID-19 provoked health anxiety and fear of meeting others in person, particularly for pwLC, and strategies to minimise face-to-face visits were implemented to limit COVID-19 spread. One strategy, telehealth, involves ‘virtual technology to deliver healthcare outside of the traditional healthcare facilities’.^32^

### Aims

1. Identify intervention guiding principles, design objectives and key features and illustrate these in an intervention logic model.
2. Conceptually test and refine intervention components, process and outcomes with service providers and stakeholders with lived experience.
3. Assess the feasibility of delivering long covid-VR and its utility and acceptability to pwLC and service providers.

## Method

### Intervention development

**Figure 1:**
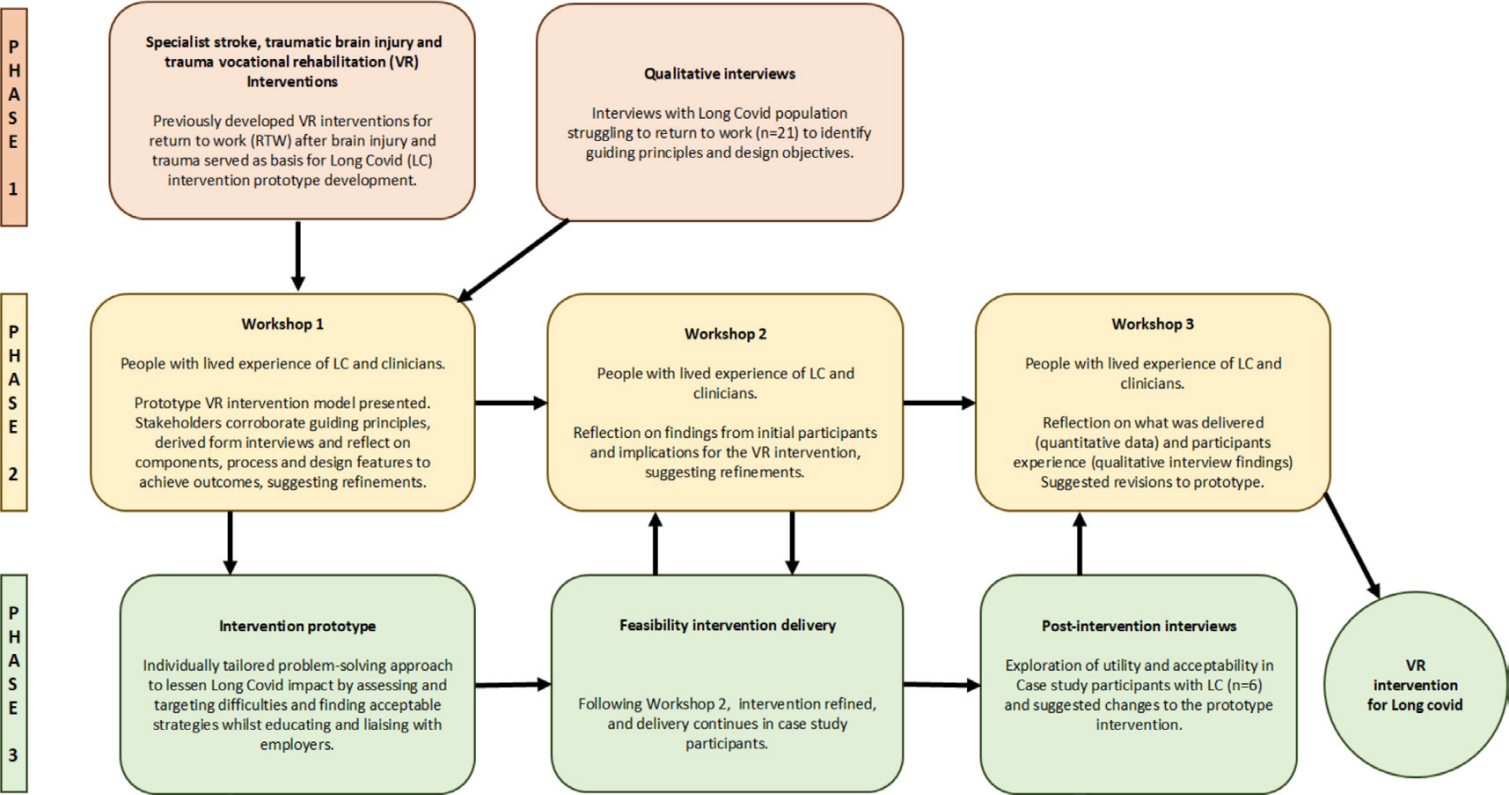
Intervention development phases.

Following guidance for developing, evaluating, and implementing complex interventions,^33^ we adopted a target population-centred person-based approach (PBA).^34 35^ This involved mixed-methods and an iterative process incorporating the views of stakeholders, resulting in an intervention that is more acceptable, contextually relevant and implementable for end users. The intervention was developed in three phases (see figure 1) and reported in accordance with the GUIDED Checklist (online supplemental material 1).^36^

Phase 1: interview study: to identify intervention guiding principles,

Phase 2: stakeholder workshops: to agree the design objectives and key features and conceptually test and refine the preliminary intervention.

Phase 3: feasibility study: non-randomised case series to assess feasibility of intervention delivery, utility and acceptability in pwLC.

### Phase 1: interview study

Issues and challenges faced in returning to work were identified in interviews exploring the lived experience of returning to work in 17 participants with long covid recruited between 21 March and 28 October 2021, using convenience sampling and a brief online eligibility survey, distributed via social media, NHS COVID-19 clinics and services, and research team networks. Participants were aged 18 and 65 years, English speaking, able to provide informed consent, had tested positive for COVID-19 and were in (self)employment, education or voluntary work at the time of infection. Importantly, they must not have returned to work or education for at least 1 month following their illness. Interviews were conducted online and analysed using framework analysis. The findings informed the intervention design objectives and a prototype intervention for discussion in Workshop 1. More details of the Phase 1 interview study are reported elsewhere.^16^

### Phase 2: stakeholder workshops

Three online stakeholder workshops were planned to obtain feedback on all aspects of design (eg, intervention components, process, duration and outcomes) and delivery; one prior to recruitment, one at a mid-point and one following intervention delivery (see figure 1). Workshops involved VR clinicians (JP, JH), members of the research team (CB, KR) and five COVID-19 patient and public involvement and engagement (PPIE) representatives. They were included in the study if they had a confirmed diagnosis of long covid, were able to speak English and gave verbal consent.

Drawing on the Phase 1 findings, our earlier RTW interventions for people with TBI, MS and stroke,^29 30^ and PPIE preferences, Workshop 1 set out to identify the intervention features to meet the RTW needs of pwLC. Phase 1 and Workshop 1 resulted in a prototype intervention for testing in Phase 3. Interim qualitative interview findings and clinician feedback from the Phase 3 testing were presented in Workshop 2 and the prototype ratified by stakeholders. Following Phase 3 testing, a description of the long covid VR intervention that was delivered (content, duration, frequency) together with findings from qualitative interviews with recipients and the occupational therapists’ (OTs’ reflections on delivery were presented to stakeholders in Workshop 3, and further design iterations suggested. For the aims and focus of each workshop, see online supplemental material 2. Members of the research team and OTs who delivered the intervention met before and after each workshop to review feedback and revise the intervention. The prototype intervention is described following the Template for Intervention Description and Replication (TIDieR).^37^

### Phase 3: feasibility study

#### Procedure

Potential case study participants were identified by members of the clinical care team from a post-COVID-19 VR team in one UK hospital and a Critical Illness Rehabilitation Class in a second. Participants were of working age (18–65 years), had tested positive for COVID-19 and were employed, self-employed or in full-time education or voluntary work at the time of infection, English-speaking, able to give informed consent and had been assessed as Level 1–6 (requires modifications) on the Work Ability Support Scale^38^ and/or had a self-identified need for support with returning to or remaining in work or self-reported ‘work issues’. People with an active mental or physical health condition causing physical deconditioning or a diagnosis of other respiratory illness were excluded. As a feasibility case series, Phase 3 did not require a sample size calculation; the planned sample was pragmatic with approximately 6–10 cases considered sufficient to inform intervention development,^31^ enable iterative refinement and explore feasibility and acceptability. This is consistent with other studies.^39^

Potential participants were identified by the clinical teams at the two sites and provided with a participant information sheet. With permission, contact details were passed to the research team, who confirmed eligibility and obtained formal written informed consent electronically using REDCap hosted at Yale University.^40^ Baseline data collection was then completed remotely by the research fellow (CB) (telephone or video via an NHS-approved platform), immediately following recruitment. Clinical work-instability assessment using the Work Instability Scale^41^ was also completed at baseline by the referring physiotherapist (ED) or OT (CK). Following baseline assessment, participants were allocated to the treating OT for the initial intervention assessment, initial vocational goal setting using Goal Attainment Scaling (GAS)^42 43^ and collaborative RTW planning. Follow-up assessments were completed remotely at 6, 12 and 16 weeks, post consent and an acceptability interview was conducted after intervention completion and prior to the 16-week assessment.

#### Baseline and follow-up measures

The measures were chosen to capture an integrated picture of participants’ recovery following COVID-19, with a specific focus on how this affected their ability to RTW. Central to this approach was the understanding that recovery from COVID-19 is not solely physical but also includes psychological, cognitive and social dimensions that can significantly impact daily function and occupational performance. The measures aimed to assess mood, anxiety, trauma-related symptoms, fatigue, cognitive functioning and the perceived impact of illness (see online supplemental material 4). Additional assessments included day-to-day functioning, perceived barriers to RTW and the extent to which long covid interfered with pre SARS-CoV-2 infection work and life activities.

Participants were asked to identify intervention goals using GAS.^42 43^ Goals were rated on a 4-point scale for importance and difficulty, where 0 is ‘not at all important/difficult’, 1 is ‘a little important/difficult’, 2 is ‘moderately important/difficult’ and 3 is ‘very important/difficult’. Following the intervention, participants self-rated the extent to which they had attained their goals (yes/no): where ‘A lot more achieved=2’, ‘A little more achieved=1’, ‘Achieved as expected=0’, ‘Unchanged=−1’ and ‘Worse=−2’.

Semistructured telephone interviews were conducted (CB) following completion of the intervention and before the 16-week assessment. Interviews explored intervention acceptability and usefulness, using a topic guide informed by the Theoretical Framework of Acceptability^44^ and the International Classification of Functioning and Health,^43^ refined by researchers (KR, CB) in collaboration with PPIE representatives. It covered topics on achievements, intervention benefits, engagement, adherence and implementation of support (see online supplemental material 3). Interviews were recorded on a password protected Olympus DS-7000 digital recorder.

### Data analysis

Individual patient data from measures of mood, well-being disability and trauma were plotted against measurement time to show possible trends from baseline to follow-up. GAS change index scores were calculated based on participants’ self-reported goal attainment, by applying the formula.^43^


$$\text{Overall GAS}=50+\;\frac{10\sum (w_{i}x_{i})}{\sqrt{0.7\sum w_{i}^{2}+0.3{\left(\sum w_{i}\right)}^{2})}}$$

where w_i_=the wt assigned to the *i*th goal (if equal weights, w_i_=1); x_i_=the numerical value achieved (between −2 and +2) and p=the expected correlation of the goal scales.

Interviews were transcribed verbatim using DICT8 (https://www.dict8.com/), a NHS Trust-approved professional transcriber, and analysed using an inductive approach to framework analysis^45^ whereby themes were generated from the data through open coding by one researcher (CB) and a third of transcripts, selected at random, second-coded (by PP) for triangulation.^46^ Codes were categorised into themes and reviewed by two researchers (JH and KR). Once themes were established and organised into a framework, transcripts were coded into the framework using NVivo V.12 (www.qsrinternational.com) by one researcher (CB) who kept a reflective journal to support analysis. The framework was reviewed by the second coder (PP) and any discrepancies discussed until consensus was achieved. The two researchers discussed the themes and their naming while reviewing the reflective notes. The results were presented to a group of three PPIE representatives with lived experience of long covid for feedback which helped to further refine themes and subthemes.

### Patient and public involvement and engagement

PPIE stakeholders with long covid were identified through an existing post-critical care PPIE group in the Nottingham University Hospitals NHS Trust and through the Trust’s PPIE lead and research team members’ networks. Two PPIE members were involved in the development and review of interview topic guides for Phase 1 and five participated in the Phase two workshops.

## Results

### Phase 1

21 participants with long covid were interviewed. Two (10%) were men. Their mean age was 41 years, range 19–58 years (SD 10.51), and 16 (77%) worked in people-facing professions. Participant characteristics can be found in table 1.

**Table 1:** Phase 1 study participant characteristics

Job	Ethnicity
Healthcare professional	White British
Allied healthcare professional	White British
Veterinary surgeon	White other
NK	White British
Allied healthcare professional	White British
School worker	White British
Healthcare professional	White other
Healthcare professional	White British
Healthcare professional	White British
NK	White British
Student	Other ethnic group
Healthcare professional	White British
Healthcare professional	White British
Healthcare professional	White British
Healthcare professional	White British
Allied healthcare professional	White British
Corporate pension actuary	White British
School worker	White British
School worker	White British
Healthcare professional	White British
Allied healthcare professional	White British

NK, not known.

Most participants experienced difficulties returning to work, particularly resulting from fatigue and breathlessness. Symptom heterogeneity from person to person and from one day to the next complicated RTW. Participants reported feeling that colleagues and managers lacked understanding, resulting in participants returning to work too soon and without adequate support, leading to relapse. A typical phased RTW was considered too short and rigid and therefore unsuitable for pwLC. Employer flexibility, demonstrated through role and task adaptations and capacity to work from home, was seen as fundamental to RTW success. Having the support of family in life outside of work was also salient for a successful RTW. The findings of this study are reported in full elsewhere.^16^ These findings were translated into intervention design objectives (online supplemental material 5) for a prototype intervention for discussion in Workshop 1.

### Phase 2

**Figure 2:**
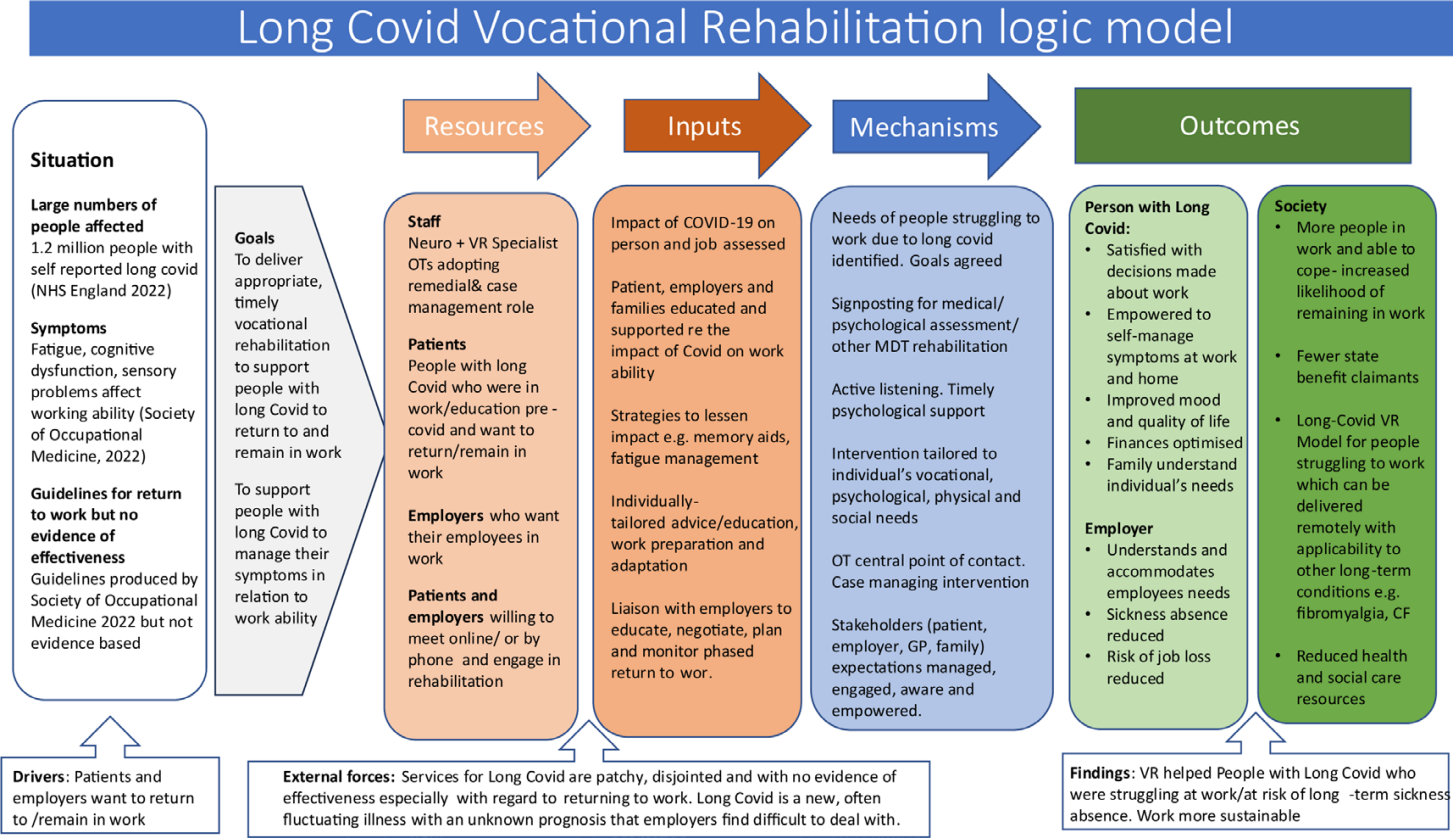
Long covid VR logic model. CF, Cystic Fibrosis; GP, General Practitioner; MDT, Multi Disciplinary Team; OT, occupational therapist; VR, vocational rehabilitation.

The intervention was iteratively co-developed in three workshops with two service provider and six PPIE stakeholders during May and September 2021 and August 2022. At Workshop 1, we presented Phase 1 interview findings, corroborating stakeholders’ experiences of important issues to address. This informed the guiding principles, design objectives and key design features of the intervention (see online supplemental material 5). Stakeholders agreed the proposed intervention process and content for the six sessions (see online supplemental material 6). The output of Workshop 1 was an intervention logic model (see figure 2), illustrating the intervention components, underlying mechanisms of change and proposed outcomes, plus any resources essential to delivery. A summary of the focus, content, decisions made and actions following each workshop, illustrating how the stakeholders and iterative feedback from the intervention delivery process shaped the content can be found in online supplemental material 2. For final guiding principles, see online supplemental table 7.

Workshop 2 reflected on the first participant’s experience and acceptability interview and the treating OT’s reflections on intervention delivery. More participant data were envisaged but ethical approval delays impacted recruitment.

Workshop 2 highlighted the need for greater flexibility in session number and format within the 12-week period. Support for managing long covid alongside work was prioritised, shifting focus from rapid RTW to realistic, gradual RTW and goal attainment. Emphasis was placed on adjusting to a new self-identity and acknowledging the slow and complex nature of recovery.

In Workshop 3, intervention delivery and interview data from six participants showed that while most had returned to work, they faced ongoing psychological challenges including adjusting to life with long covid, processing traumatic experience and performance and symptom anxiety. All had more than the pre-planned six sessions and were referred for additional help at the end of the 12-week intervention. Further details of the intervention delivered are reported in Phase 3 and online supplemental material 7. Stakeholders recommended extending the intervention duration, increasing session numbers and incorporating referrals to other professionals (eg, psychological support, physiotherapy for breathing exercises). They stressed the importance of ongoing monitoring, review and coordination to support sustained work participation.

Following the final workshop, the intervention was redesigned as a longer-term, job retention-focused intervention. It included strategies for managing long covid in the workplace, educating employers and colleagues and negotiating tailored accommodations. This marked a shift from short-term RTW preparation to a more holistic, sustainable approach.

Reflecting on whether the intervention was a Tier 3 (specialist) intervention, and whether it could be delivered by therapists without specialist VR training, OTs concluded the intervention was Tier 3. This was due to the longer-term, complex nature of participants’ problems, red flags, the need for more nuanced support, often more than 6 months after infection and the specialist knowledge and skills required,^8^ including fatigue management, identity support and how to negotiate with employers and line managers. Knowledge of the Equality Act^47^ and Health and Safety legislation and schemes like Access to Work, a UK government scheme to help people with disabilities remain in work,^48^ was essential for effective delivery.

### Prototype intervention

The proposed long covid VR intervention assumed a problem-solving approach based on an underlying holistic biopsychosocial model of illness, the International Classification of Functioning and Health (WHO, 2001),^49^ the Work Disability Paradigm^50^ and theories of behaviour change.^51^ The intervention seeks to lessen the impact of COVID-19 by assessing the patient’s role as a worker and finding acceptable strategies to overcome problems directly impacting work activities (eg, physical, cognitive, psychological or task/environment-based). The underpinning programme theory supporting the intervention is outlined in online supplemental material 5.

**Figure 3:**
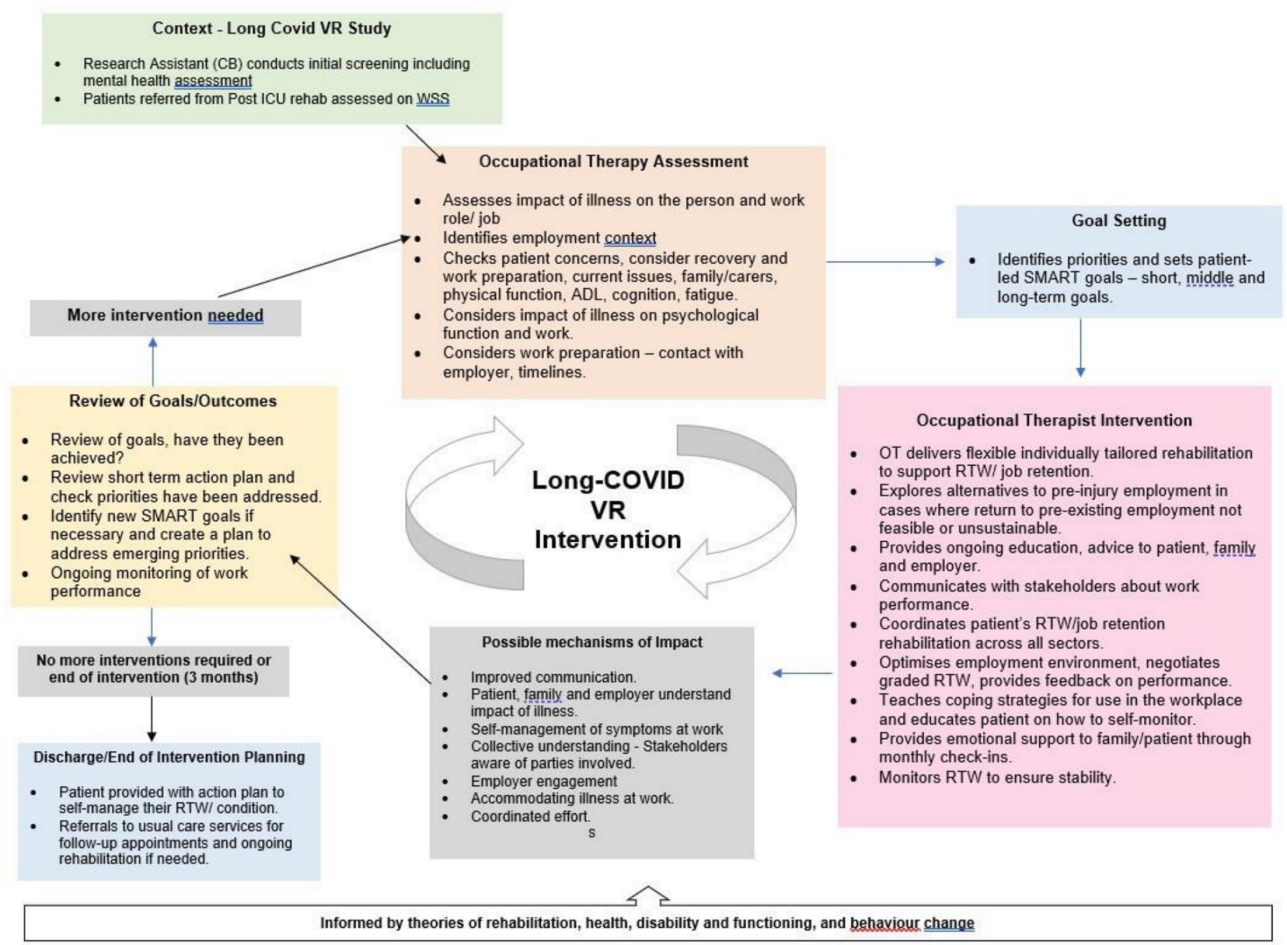
Long covid VR intervention process. OT, occupational therapist; RTW, return to work; VR, vocational rehabilitation; ADL, Activities of Daily Living; ICU, Intensive Care Unit; SMART, Specific, Measurable, Attainable, Relevant, Timely; WSS, Work-ability Support Scale.

The long covid-VR intervention adopts a cyclical process (see figure 3), involving an assessment of the impact of COVID-19 on the patient and their job, vocational goal setting (and review) and an individually tailored programme. This includes education for patients, employers and families about the COVID-19 impact on work; identification of strategies to lessen impact, for example, memory aids, pacing to manage fatigue; work preparation, that is, helping the patient to reestablish routines with gradually increasing activity; seeking opportunity to practice work skills, for example, computers to increase concentration, walking to increase stamina and liaison with employers and employment advisors to negotiate, plan and monitor a phased RTW.

The intervention was intended to be individually tailored in content, dose, intensity and duration according to participants’ needs and preferences (eg, whether the participant consents to employer engagement and remote workplace visits or only accepts advice about employer liaison) and employment context (eg, where there is no employer to liaise with or participants RTW early and are unable to meet the therapist in person at the intended frequency, resulting in online or telephone intervention). It was designed for remote (online) one-to-one delivery via MS Teams/other NHS approved platform by an OT. In addition, liaison with or about the participant might involve email, telephone (calls or texts) and online meetings with others such as workplace representatives (eg, OH, line managers, human resources services) or other health or social care professionals with the participant’s consent. Meetings might typically involve RTW planning, negotiating phased return, workplace accommodations (changes in hours or responsibilities or supernumerary support as needed), to enable the participant to RTW and remain in work. Intervention sessions were designed to last for approximately 1 hour. We anticipated approximately six face-to-face contacts/participant over 12 weeks with the number of sessions determined by participant need. At the initial OT assessment, the participant and OT agreed the focus and planned components of COVID-19-VR (including prioritised goals, anticipated session frequency, and whether employer contact/meetings were permitted). Participants were informed that the programme would run for approximately 12 weeks with an anticipated average of six sessions, but that session number and mode of contact (video/telephone and non–face-to-face liaison) could be flexed in response to need and work context. Prior to the initial assessment with the OT and as part of the Phase 3 study baseline assessment, participants were screened for mental health problems using standardised assessments of mood (Patient Health Questionnaire (PHQ-9)^52^ and Generalised Anxiety Disorder (GAD-7)^53^) and referred for psychological support as needed in consultation with a psychiatrist (RM) and clinical psychologist (RdN). Those identified with mild to moderate mental health problems were referred to the General Practitioner or local talking therapies (eg, Improving Access to Psychological Therapies service). The prototype intervention is described following TIDieR in online supplemental material 7.

### Phase 3

#### Participants

**Figure 4:**
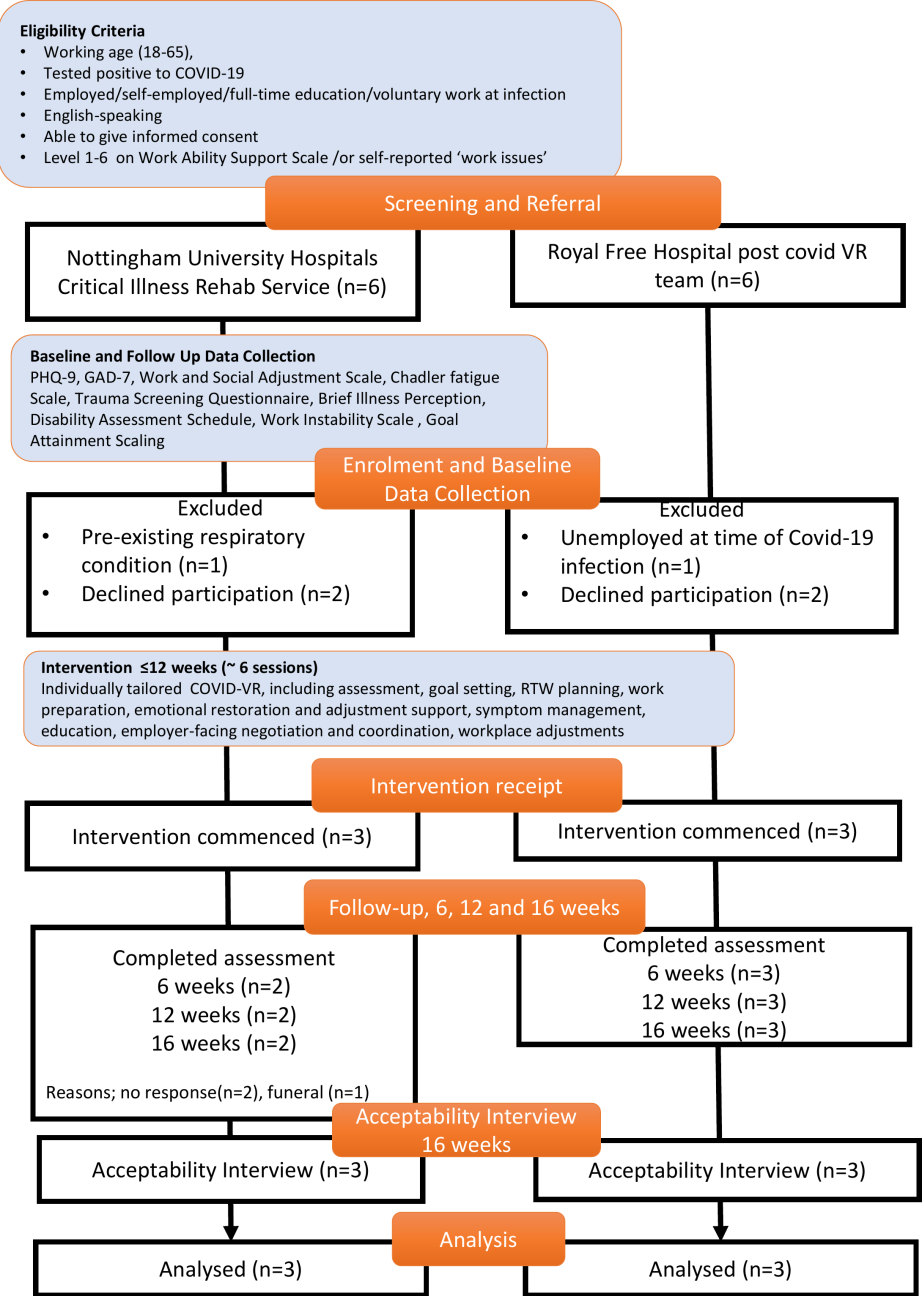
Phase 3 study flow diagram. GAD-7, Generalised Anxiety Disorder-7; PHQ-9, Patient Health Questionnaire; RTW, return to work; VR, vocational rehabilitation.

Of 12 potential participants identified, four declined participation and two failed to meet the criteria because of a pre-existing respiratory condition (n=1) or were unemployed at the time of COVID-19 infection (n=1), see figure 4. Six participants were recruited between 26 May 2021 and 21 March 2022.

Participants were aged 18–64, mostly female (66.7%) and ethnically diverse, identifying as Asian/Asian British (33.3%), mixed/multiple ethnic groups (33.3%), white (16.7%) and African/Caribbean/black/black British (16.7%). Most (83.3%) reported no disability, and one held a higher degree, and four were married. Time between COVID-19 infection and the intervention commencing ranged from 5 to 24 months (mean 13.3 months). Two participants were under investigation for additional conditions (back pain, glandular fever, lymphadenopathy, recurrent urinary tract infections). Three participants were admitted to intensive care with COVID-19 infection, and three were primary care referrals. All (three from each site) received the intervention as intended between June 2021 and June 2022. The mean time between COVID-19 onset and the start of the intervention was 13 months (range 5–24 months). See table 2.

**Table 2:** Phase 3 participant characteristics

Pt ID	Age	Pre-COVID-19 occupation
1	55–64	Sales
2	18–24	Student
3	45–54	Teaching assistant
4	45–54	Teacher
5	45–54	Teaching assistant
6	45–54	Healthcare technician

#### Follow-up

Of the six participants, five completed the follow-up assessment at each time point. One participant was missing at each follow-up (see figure 4). All participants consented to participate in the follow-up interview.

#### Work status

The pre-COVID-19, pre-intervention and post-intervention work status of participants are shown in online supplemental material 11. All participants were in full-time work or education before COVID-19 infection. At the start of the intervention, five had returned to work or education but reported that they were struggling. Following the intervention, five remained in work, one participant increased their hours, two decreased their hours, two retained the same hours. One participant who was working full time at the start of the intervention applied for a reduction in working hours to 60% WTE instead of retiring. One participant who was not working at the start of the intervention was undertaking a voluntary 4-week unpaid work trial of 2 hours/day to determine readiness for a formal graded RTW by the end of the intervention.

#### Additional outcomes

No changes were observed between baseline and follow-up on measures of fatigue, disability, illness perceptions, generalised anxiety or trauma. However, changes in group median scores were observed on the Work and Social Adjustment Scale^54^ and the PHQ-9,^52^ indicative of improvements in mood from moderate to mild depression, and improvements in symptoms associated with functional limitations or psychopathology affecting work and social participation. See table 3.

**Table 3:** Median, minimum and maximum for each outcome measure over timepoints

	Median (min–max)							n
	PHQ-9	GAD-7	WSAS	TSQ	CFS	BIP	DAS	
Baseline	13.00 (8–17)	11.00 (5–16)	28.5 (18–36)	3.50 (2–7)	7.50 (6–9)	61.0 (57–67)	24.50 (12–31)	6
6 weeks	9.00 (5–16)	6.00 (3–12)	31.00 (8–32)	4.00 (0–6)	7.00 (6–8)	60.0 (54–64)	24.00 (17–28)	5
12 weeks	8.00 (0–14)	4.50 (0–8)	21.50 (0–31)	4.00 (0–8)	7.00 (0–9)	52.5 (0–61)	19.00 (4–33)	5
16 weeks	7.00 (5–10)	10.00 (4–14)	19.00 (7–30)	3.00 (0–6)	7.00 (7–7)	54.0 (51–57)	24.00 (9–29)	5

BIP, Brief Illness Perception (scores range 0–80, lower scores are better); CFS, Chader Fatigue Scale (scores range 0–33, lower scores are better); DAS, Disability Assessment Schedule (scores range 12–60, lower scores are better); GAD-7, Generalised Anxiety Disorder 7 (scores range 0–21, lower scores are better); n, number; PHQ-9, Patient Health Questionnaire (scores range 0–27, lower scores are better); TSQ, Trauma Screening Questionnaire (scores range 0–10, scores 6–10=possible PTSD); WSAS, Work and Social Adjustment Scale (maximum score 40, lower scores are better, scores over 20 suggest moderately severe or worse psychopathology).

#### Intervention delivery

In total, the six participants received 51 intervention sessions (range 6–13) (median 8; IQR 7–10), totalling 142 hours of OT over a median 81 days (IQR 73–82, range 45–85 days). Each session lasted approximately 65 mins (range 15–123). The total amount of face-to-face delivery time was 55 hours, range 6.35–13.83 hours (median 9.2, IQR 2.15–7.38). Non-face-to-face time totalled 87 hours, range 0.16–3 (median 13, IQR 9.38–1.68). This included time sourcing information, writing session summaries, completing Allied Health and Work reports,^55^ referring to other healthcare or government services (eg, access to work, and coordination with others (ie, carers, employers, health professionals)).

All participants received VR for 12 weeks. Time between consent and intervention starting ranged from 1 to 8 days. The intervention was intended to be delivered weekly, but this varied due to individual circumstances. The mean number of days between sessions ranged from 1 to 14 days.

Dealing with ‘current issues’ consumed the most time (eg, claiming disability benefits, such as Personal Independent Payment and Universal Credit, resuming previous activities, discussing COVID-19 experiences). These were issues deemed important by participants but not considered VR. See table 4. Dealing with fatigue, breathlessness and psychological issues consumed similar amounts of time. Only one OT had contact with participants’ employers. No time was spent on helping participants find alternative work, assisting with mobility or on other activities of daily living. Time spent on ‘cognition’ included identifying and determining the impact of and educating participants about cognitive problems (such as attention, memory, planning, organisation and multitasking) and finding acceptable strategies for managing them in the workplace. No participants were referred for psychological support. However, one person was supported to activate an existing open access appointment. See online supplemental material 7 and figure 3 for more details of the intervention.

**Table 4:** Total time spent on intervention components across participants

	Most delivered component	Time (min)	Least delivered component	Time (min)
1	Current issues	410	Job redirection	0
2	Fatigue, pain, breathlessness	385	Other	10
3	Psychological issues	307	Psychological screening and referral	10
4	Work-related assessment	240	Mobility	45
5	RTW with direct contact with employer	235	Activities of daily living	57
6	Goals	230	Family/carer support	85
	Monitoring job retention	229	Physical	91
7	Work preparation	205	Cognition	145
8	Dealing with medical issues	195	RTW without direct employer contact	160
9	Homework	190	Assessment	170

RTW, return to work.

#### Participant goals

Each participant set goals that were important to them, but all ranked them as ‘difficult to achieve’. Following the intervention 10 of the 17 goals were rated as achieved with 4 ranked ‘a lot more achieved’ and 5 ‘achieved as expected’. Three were ranked as ‘partially achieved’ and one the ‘same as baseline’. All participants achieved at least one goal. GAS goals and change scores are shown in online supplemental materials 8 and 9.

### Acceptability and utility

Interviews lasted between 12 and 46 min. Qualitative analysis revealed an overall picture of the intervention’s effectiveness across three themes and nine sub-themes (table 5). Emotional restoration (theme 1) emerged as a pivotal dimension, with empathetic listening, understanding and emotional support playing key roles in enhancing participants’ mental well-being and motivation to RTW. Participants reported gaining valuable insights into their symptoms and learnt adaptive strategies, empowering them to navigate their daily lives more effectively. The intervention also provided ‘Resources and practical benefits’ (theme 2), including access to essential tools for symptom management and facilitating the transition back to work. Central to its success was its unique service configuration (theme 3), with a focus on a collaborative approach involving tailored RTW plans and effective communication, negotiation and education of the participants’ employers. Despite initial apprehensions, participants overwhelmingly found the intervention invaluable, underscoring its importance in their RTW journey. While remote delivery offered flexibility, participants expressed a preference for face-to-face sessions as they felt more able to express how they felt. Themes are summarised and illustrated in table 5.

**Table 5:** Summary of themes and subthemes

Theme	Subtheme	Definition
Emotional restoration	Listening, understanding and emotional support	Therapeutic effects from being listened to empathetically and understood in a personalised way, contributing to improved mental well-being and RTW motivation.
	Understanding symptoms and self-acceptance	Gaining insight into long covid symptoms, leading to acceptance and informed adaptation of work or life routines.
Resources and practical benefits	Information providing	Empowering participants by clarifying complex systems (eg, benefits, health services, work rights) and assisting with access through referrals and advocacy.
	Tools, techniques and symptom management	Learning specific strategies (eg, pacing, rest, planning) to manage cognitive and physical symptoms, helping regain daily function and prepare for RTW.
Service configuration	Work accommodations for RTW	Implementation of tailored RTW plans including adjustments like phased returns, modified duties or support services.
	Employer education and negotiation support	Facilitating communication with employers to explain participants’ needs and negotiate appropriate workplace adjustments through reports and joint meetings.
	Participant experience and session flexibility	Participants’ reflections on the intervention’s benefits, the therapeutic relationship and desire for more flexible and prolonged access to support.

RTW, return to work.

#### Theme 1: emotional restoration

The intervention positively impacted participants’ mental well-being, which was an important part of the intervention.

##### Listening, understanding and emotional support

Listening to the participants and their specific situation and symptoms, while demonstrating empathic understanding such that they acknowledged the participants’ difficulties, had therapeutic effects. The emotional support experienced related to the overall situation, including mental health and bereavement (losing a close family to COVID-19). Understanding the patient’s background was important for tailoring the rehabilitation. Twinned with regular sessions, the understanding and listening increased participants’ overall motivation and motivation to RTW.

It’s really important the professional listened to my circumstances, and I didn’t feel at any time I was getting […] a one solution fits all, to listen to the problem, I really felt that it was about what worked for me. (Participant 5)

You know when you have got somebody there that it is going to come back to you after a while and say, ‘Look let’s see what you have achieved with forward planning, what you have done’ and that’s what I feel makes you, gives you that confidence and that inspiration to move on and do things. (Participant 1)

##### Understanding symptoms and self-acceptance

Through the intervention, participants gained knowledge about their life-changing long covid symptoms. They had to relearn their work, adapt to symptoms and even consider changing role. Recognising and accepting their new situation through specialist advice was salient for a sustained RTW.

I think it has honestly been invaluable for helping me recognise that I was quite significantly ill and I needed to acknowledge that first before I could work out what work I could do and how I could gradually increase those hours of work and with a plan to being back full time. (Participant 5)

It has helped my work-life balance, […] it is also a stamp, an external sort of verify to say well actually am I doing too much, do you think you need to back down and take a step back. (Participant 6)

### Theme 2: resources and practical benefits

The intervention was educational, in that they gained practical knowledge and learnt symptom management techniques, thereby improving their day-to-day life.

#### Information providing

Participants accessed information about benefits, policies, health procedures, services and schemes, which were perceived as confusing and hard to access prior to the intervention. OTs helped participants access financial support services and schemes to commute to work (Access to work,^48^ or understand their employment rights). Accessing such knowledge was empowering for one participant. Information-providing extended to liaison with other professionals and services through referrals, following up and supporting participants to manage appointments.

[The OT] was able to contact [other] services, check in, work out where I was on the waiting list, explain this to me […] when you’re unwell, that all becomes quite overwhelming and hard to do. […] It was empowering me to know my rights in the workplace and to slightly assert those rights in meetings with management. (Participant 5)

When I tried to get advice from Social Services […] it was awful, you ring numbers up and I was left on hold for 45 min to an hour, sometimes when you did get through you were told you were at the wrong Department, and they couldn’t transfer you, so you’d have to ring back again. (Participant 3)

#### Tools, techniques and symptoms management

The intervention helped participants manage their symptoms through providing and explaining exercises and techniques. Participants particularly noticed improvement in dealing with cognitive symptoms (eg, memory, focus) and monitoring sleep and fatigue while also acknowledging the usefulness of general guidance (eg, work-life balance). They felt that concepts such as rest were not well explained by other healthcare professionals, making the intervention educational and valuable. Managing symptoms was linked to returning to a routine and reconditioning—important RTW preparations. However, one barrier to accessing certain suggested symptom management classes was that they were only available during working hours.

Before I was going to [return to work, the OT] prepared for it by doing things at a certain time of the day and being organised, to get my mind and my body ready for that two- or three hour session of work, so [the OT] gave me the tools to prepare myself initially for that. (Participant 3)

Rather than stopping when I was tired and to take a break […], to stop before we got to that point because it was quicker then to recover […]. To take a break, it doesn’t mean going watching a video or YouTube or anything like that, rest means just having a complete break. (Participant 4)

The physio gave me some advice and stuff to help me with the fatigue, but the thing is everything they suggested like you know yoga classes and other, it was all in the morning and I am at work. (Participant 2)

### Theme 3: service configuration

The intervention helped to elaborate and communicate a RTW plan with the participants’ employers.

#### Measures and changes to work to facilitate return

Participants felt confident in the plans, which included reasonable adjustments and tailored phased RTW. The measures ranged from role-specific support, such as access to support staff provided by the employer (eg, appointed assistant), support with the commute to work and starting with less demanding tasks, to taking several breaks throughout the day.

I feel very confident that the plan that we have in place now is the correct prolonged return, phased return because it’s realistic. (Participant 5)

[The OT] gave me the taxi service I think, somebody would pay for my taxi to and from work. […] Normally, I am customer serving and a very stressful area of the business and so [The OT] advised to start off out of the way in the back office with another staff member (Participant 2)

#### Employer’s education, negotiation and compliance with RTW plan

A crucial part of the intervention was engaging with employers about participants’ situation, symptoms and their effects on work. Communication took the form of reports, letters and meetings. The latter were perceived as valuable for ensuring the participant was not agreeing to tasks they could not manage and negotiating a RTW plan, relevant support and adjustments. The plan and guidance were perceived as relevant by employers as they ‘came from a specialist’. While some employers took the guidance on board, some lacked funding for the requested support or insisted on employer-based OH input.

Sitting in on the Teams meetings with my work HR and the Management Team. [the OT] has been helpful with getting me ready for that and also with being on board with meetings and asking questions really that I wouldn’t have thought of asking. (Participant 3)

I wouldn’t have had half the confidence in those negotiations without the support of the occupational therapist. […] it was acknowledged by (my employer), that my occupational therapist had a better understanding of where I was (Participant 5)

[My employer] said no they can’t do anything about [the OT’s suggestions] because it has to come from our own Occupational Health (and that) they don’t have extra money for extra staff. (Participant 2)

#### Participants’ experience with, and impression of the intervention

Although apprehensive before their first session, all but one participant found the intervention beneficial. Attending the sessions gave them purpose. Flexibility around fatigue was positively acknowledged, for instance when the OT noticed tiredness and ended or rescheduled the session if necessary. The consensus was that more sessions would have been beneficial until return to good work, or the possibility of accessing the OT when needed until that point (eg, special meeting or events requiring further support).

I was a bit apprehensive when it all began but yes definitely sleep wise and relaxation wise it helped me, clinical wise, definitely helped me most with the cognitive and the thinking side of things. (Participant 3)

As soon as I found it hard or I got a bit foggy brained or my concentration was waning or whatever, we stopped the sessions. [However] there’s always the worry that I might not get access to the other services or that I feel worse again and might need the support of someone who is aware of my rights that if I did feel worse again. (Participant 5)

#### Remote delivery

Although participants only occasionally experienced minor difficulties such as being on mute while speaking, videoconferencing was seen as possibly restrictive. Participants felt face-to-face delivery would have better enabled them to express their feelings, as the virtual room was not perceived as ‘real’ and family members could eavesdrop. Moreover, participants believed that in-person sessions could have enabled practical activities (eg, walk and talk, exercises), beneficial to long covid recovery. They were not concerned about COVID-19 infection risk linked to face-to-face sessions. However, remote delivery offered some advantages; it was more flexible and convenient to fit around work, cheaper and effective for managing fatigue. Altogether, it was recognised as down to personal preference, with those advocating for face-to-face treatment acknowledging that videoconferencing did not affect their relationship with the OT, which was important, given the seriousness of the topic of conversation.

You know when you are talking on zoom, you are not always sort of like yourself and you just might sort of like get along and say, Yes, I am here, OK [and], you don’t know whether [family members] are peering over the door, or just listening and sometimes you may want to say something and you don’t want them to hear. (Participant 1)

I think had it been a face-to-face, probably would have been just as tired to be honest as well and the effort to be able to go out. (Participant 4)

A couple of times I forgot to switch the volume up so they couldn’t sort of hear me etc so I think it was more user error than anything else [but] I think we still managed to have a good relationship, we never missed any meetings. (Participant 3)

#### OT experience and characteristics

The OT, as a clinician, had an impact on the intervention. Participants commented on the importance of the OT’s background and experience and their ability to link symptoms to existing research and find solutions. Communication factors, such as being prompt and emailing session summaries and exercises, were perceived as helpful. Building a strong relationship with the OT was salient to the success of the intervention.

[The OT] would always go away and do some research and come back, well either e-mail me back with information or give me some more information the next week we came back in, so it was really helpful from the start. (Participant 3)

This is quite important, I felt that my OT was very knowledgeable in these things as well. (Participant 6)

#### Assessing readiness to RTW

Participants were asked how to best measure readiness to RTW. Having an ongoing conversation was deemed the most appropriate way to assess the person and their situation, reflecting on the impact of increasing hours and reducing them to an acceptable level if found to be too much. However, returning to the actual job as soon as physically ready, to assess difficulties in the workplace was also mentioned.

The only way you can really measure is possibly by ongoing conversations with the people you are talking to see whether their phase, all sort of ending at the same stage mine did, where you just started to go back to work for the early stage of the phased. So the only way to measure it is to say conversation with them on a couple of months or so and just see whether they have been able to keep graduating up their level of hours or days or whether they have not been able to. (Participant 3)

I think the best way for me was to just put myself in at the deep end and see how I could manage because there is no other way of knowing how much I can do. (Participant 6)

## Discussion

Following the MRC Framework for developing and evaluating complex interventions,^33^ we used the PBA^35^ to develop an intervention to address the VR needs of pwLC. To our knowledge, this is the first VR intervention systematically developed using PBA to support pwLC to return to and remain in work. Triangulation between in-depth qualitative research and the intervention users’ and PPIE members’ lived experience reassured us that the intervention components and mechanisms are relevant and likely to influence positive outcomes for this population. Reflecting participants’ experience of the intervention (phase 3) against the lived experiences of pwLC (phase 1) and feedback from PPIE and therapists involved in intervention delivery (phase 2) enabled us to co-develop and refine our logic model and guiding principles. The intervention now needs to be refined following initial implementation and tested in a larger group of pwLC. While guidelines and tiered pathways outline broad principles for supporting work participation, COVID-19-VR operationalises these into a deliverable, specialist job-retention programme with explicit components (eg, structured vocational goal setting, symptom-management in a work context and employer-facing negotiation and coordination).

What COVID-19-VR offers is a specialist, job-retention VR package for pwLC that translates broad ‘return-to-work advice’ into concrete clinical actions and service processes. Developed iteratively using a PBA, the intervention combines (1) emotional restoration and adjustment support (empathic listening, validating fluctuating limitations, supporting self-acceptance and confidence), (2) practical resources and symptom-management tools tailored to working life (eg, pacing/rest planning, cognitive strategies, preparing for work routines) and (3) a distinctive service configuration in which the OT actively coordinates and negotiates with employers, using written reports and joint meetings, to secure realistic adjustments and prolonged, personalised phased returns. Feasibility testing also clarified key implementation parameters: the need for flexibility in session number and format, frequent non–face-to-face coordination time and access to multidisciplinary input for some participants (eg, psychological support).

Findings from this and our previous study^16^ indicate that long covid exacerbated participants’ RTW. The intervention’s primary goal was job retention (returning to an existing job or role), which involved engaging with and negotiating workplace accommodations with patients’ employers to enable people to cope with job demands.

The intervention predominantly focused on helping participants recognise, accept and explore ways to manage symptoms at work. Consistent with other research,^8^ breathlessness and fatigue most affected workability. However, participants also had high pre-intervention levels of depression and anxiety, and while mood improved for some, anxiety levels remained high following the intervention. Participants expressed frustration regarding employers’ lack of acknowledgement and understanding of their condition. However, at the time of intervention delivery, long covid was not a recognised condition, and little was known about the long-term manifestation of symptoms or its impact in the workplace. This may have changed now, given the increased awareness and public discourse around long covid.

The effect of the pandemic on the VR is important to consider. While fatigue and breathlessness presented the greatest work barriers, most intervention time was spent on immediate issues, such as information or support for claiming state-provided benefits and informal psychological support (two participants had close relatives die of COVID-19), which were not directly work-related. These issues needed addressing before participants could engage in VR and focus on RTW. This may reflect recruitment during the pandemic when national lockdown measures disrupted people’s lives, resulting in high anxiety levels^56 57^ in addition to disease-specific uncertainties. Therefore, a holistic view of the patient’s situation and difficulties beyond symptoms was needed. This differs from other COVID-19 RTW interventions predominantly targeting long covid symptom management.^25 26^

Overall, participants found the VR useful; they learnt to manage their symptoms and instigate conversations with their employers. However, not everyone returned to work by the end of the 12-week intervention, and some who were working were struggling, suggesting some people may need more support or a different kind of intervention.

Participants stressed the importance of communicating with their employer regarding symptoms and role adjustments that went beyond the ‘standard graded RTW process’ requiring OT-supported negotiation, translation of symptoms into functional constraints and ongoing coordination with employers. These findings align with a recent review on RTW,^58 59^ which identified that a lack of employer understanding about the condition may lead to unrealistic expectations. In COVID-19-VR, this was addressed through employer-facing education and structured liaison (with participant consent), including OT-led communication and joint meetings to negotiate realistic adjustments and phased returns.^58^ In our study, the OTs acted as coordinators, facilitating communication between the patient, the employer and the RTW process^58^ and assumed the role of negotiator,^60^ recognising that while the employer holds a power advantage in decisions on adjustments, the clinician can negotiate the terms of the return using their medical expertise and knowledge of legislation (eg, in the UK, The Equality Act, 2010). Those receiving the intervention felt their employer was more likely to listen to the clinician who also offered support during meetings.

Adopting a negotiator role may mean that clinicians delivering long covid VR require additional specialised training. In this study, both OTs were VR experts. In exploring their perceptions of the intervention’s fit with NHS England’s proposed three-tier model and the reasons that prompted primary care referrals for specialist VR support at one of the sites, this study highlighted three primary instigators. The need for ‘legislative knowledge’, the ‘OTs’ employer negotiation skills’ and managing uncertainties pertaining to long covid such as its fluctuating nature and red flags, which warranted further investigation or input from other members of the multidisciplinary team. These findings suggest the intervention fit with a specialist Tier 3 long covid VR intervention (see online supplemental material 10).

Some participants had already returned to work but reported difficulties linked to their long covid symptoms. Thus, the intervention focused on finding ways of coping with work as opposed to preparing people to RTW. This differs from existing research whereby most participants were in employment but had not managed to RTW.^26^ This may be important in terms of the VR intervention content. Participants who have not returned to work are likely to experience stronger symptoms resulting in greater focus on symptom management. Whereas in this study, negotiating adjustments was more relevant. Moreover, much of the rehabilitation was targeted toward helping participants accept what they could and could not do. Indeed, accepting that they may be unable to resume their pre-COVID-19 role without adjustment or modified duties has been identified as a predictor to RTW.^26 61^

Interestingly, participants’ goals changed over time as they realised their limitations and became more realistic about what could be achieved in the time available. For example, ‘returning to work as soon as possible’ became ‘preparing to RTW’. One participant realised her goals ‘to stop doing certain duties at work’ and ‘change others’ attitudes’ were beyond her control. Uncertainties related to long covid itself at the time of study made it difficult for both the patient and OT to set realistic goals, as there was little in the way of pattern recognition by drawing on previous cases or experience when dealing with symptoms or work-related problems^62^ for the OT or lived experience for the patient. However, as with other conditions resulting in chronic fatigue, the OTs rapidly recognised boom-and-bust cycles and the need for the patients to accept their diagnosis, recognise symptoms and current limitations, so that conversations about their impact on work abilities could occur between the patient and employer and reasonable adjustments that allow for more sustainable work could be negotiated.^63^,^65^

The intervention we developed appears to support individuals in different ways, depending on their needs and context. It incorporates both restorative and adaptative or compensatory elements. For example, the use of activities to build stamina and concentration in preparation for work return (restorative) and the use of compensatory strategies to support memory difficulties or adapt the job, role or responsibilities to accommodate long covid disability. However, considerable time was also spent in helping patients accept and come to terms with the chronic, long-term, uncertain and fluctuating nature of the symptoms, especially breathlessness and fatigue, which are very debilitating at work. The OTs not only required specialist knowledge and skills in goal setting, employment legislation and employer negotiation, but also in fatigue management and providing emotional support.

The varied, complex and uncertain nature of long covid necessitates an individually tailored approach. The persistent, fluctuating and evolving needs associated with long covid far exceeded our initial expectations of short-term advice and support to prepare people to RTW. These needs align closely with those observed in individuals managing long-term neurological^29^ or mental health conditions,^66^ emphasising the importance of (re)accessible and ongoing support, help with adjustment, and psychoeducation.

The unpredictable ways long covid manifests across individuals contribute to significant uncertainty about the legacy of COVID-19 and its long-term impact at individual, health service and economy levels and underscore the urgency of addressing the employment needs of those with long covid both now and in preparation for future pandemics.

### Strengths and limitations

A key strength is the stepwise, stakeholder-informed co-construction of COVID-19-VR using the PBA, maximising alignment with pwLC needs and real-world delivery constraints. This was a small study, and participants may be atypical of the wider population living with long covid. Most were infected before vaccinations became available and three were hospitalised and ventilated in a critical care unit. Four were also employed in teaching or healthcare professions, in roles that were difficult to adapt and which were deemed essential or core work during the pandemic.^67 68^ Most presented with severe long covid symptoms and most (four) were also women, possibly having to contend with homeschooling alongside work. However, this deep dive into understanding the vocational support needs of these pwLC during the intervention development process is a potential strength of this study, as it encompasses and highlights some of the most challenging contextual factors likely to affect wider clinical implementation. As a small, non-randomised case series, we cannot estimate intervention effectiveness or superiority. Furthermore, observed work outcomes may reflect natural recovery, workplace context, or concurrent supports, and the sample size was intended for feasibility and intervention optimisation rather than effectiveness testing.

### Clinical implications

It is possible that different types of intervention are needed; one targeted at preparing people to RTW and one specific to those who are in work but struggling with long term, fluctuating long covid symptoms, such as fatigue and high levels of anxiety that threaten work stability.

The intervention was originally designed for independent, uni-discipline (by a single OT) online delivery over six to twelve weeks. However, all participants used the full duration of the intervention, and most commented that they would have benefited from further support. The persistent, fluctuating and variable nature of the long covid symptoms over time mean that people may require support extending beyond 12 weeks, particularly those hospitalised following SARS-CoV-2 infection. This could take the form of regular check-ups or top-up sessions, according to need. Participants also reported the need for the presence of the OT during meetings with the employer. Therefore, it would have been useful to have this continuity and support during those meetings as a minimum.

Some participants also required the input of more than one type of healthcare professional and despite a reluctance for referral to a psychology service, some had persisting emotional difficulties warranting psychological intervention. These findings suggest the need for access to a multidisciplinary team and individual or group-based psychological support as an optional bolt-on to the intervention. Such a mixed model has been successfully delivered as part of VR interventions for other patient groups, for example, survivors of major trauma.^31^

## Conclusions

PwLC may need specialist help to RTW. Our long covid VR intervention was feasible and acceptable in this small case series. This is perhaps because the intervention was iteratively developed following a PBA, which inherently supports co-creation. The study also highlighted substantial implementation demands (eg, coordination time) and identified candidate mechanisms (eg, workplace negotiation, emotional support and symptom-management strategies) that warrant explicit testing. These findings support further staged evaluation to refine outcome selection and assess efficacy and initial implementation in a larger sample, prior to any definitive trial.

## Supplementary material

10.1136/bmjopen-2025-109740 online supplemental file 1

10.1136/bmjopen-2025-109740 online supplemental file 2

## Data Availability

Data are available upon reasonable request.
