# Supplementary material for: Developing a job retention vocational rehabilitation intervention for people with long covid: a person-based approach
Source: BMJ Open. 2026 May 15;16(5):e109740. doi: 10.1136/bmjopen-2025-109740 (PMC13182320; doi:10.1136/bmjopen-2025-109740)
Supplement: online supplemental file 1 [file bmjopen-16-5-s001.docx]

# Supplementary Material

**Supplementary Material 2: Focus and Actions of Workshops**

| **Workshop and Stakeholders** | **Focus** | | **Stakeholder feedback; decisions made and agreed actions** | | |
| --- | --- | --- | --- | --- | --- |
| Workshop 1 (June)  People with lived experience of LC (n=4) and clinicians (n=3). | - Aims of the study and purpose of workshops - Presentation of Long COVID related work issues identified in pre-intervention interviews.   - Participants asked to corroborate interviewee experiences/ work-related issues and asked about support they would want. - Review proposed Intervention outline and session content and structure (pre-delivery)   - Participants asked What they would want the OT to do/ask them/talk about; online delivery challenges; suggested changes to proposed intervention. - Considerations for feasibility study. | | **Intervention should (Guiding Principles);**   - Talk about time frame for recovery - Provide - information and guidance to employers about Long COVID – including, fluctuations, recovery expectations and mental health effects – ‘Long COVID is too new and employers do not know what to do’. (Employers need to provide support for mental health (especially for critical care patients). - Provide - individually tailored Phased Return to work - Understanding a phased return to work with Long COVID can be challenging. ‘Be as creative as possible with the return to work (RTW) plan’ - a much longer process and tailored return may be required dependent on symptoms. - Offer psychological support (or refer to an appropriate service) – and psychoeducation regarding the autonomic nervous system and benefits of relaxation. The physical manifestations of anxiety are like Long COVID symptoms. Anxiety is secondary to Long COVID and so OT should focus on differentiating these. - Offer stress management as well as fatigue management, as stress can exacerbate the symptoms, pushing the autonomic nervous system into overdrive (participants suggested exercise classes, creative activities and socialising) - Assess and screen for red flags, triggering referrals back to local Long COVID clinics to obtain medical perspective on worrying symptoms, as many GPs not actioning these referrals (during pandemic).   **Recommended resources and bolt on activities.**   - <https://stasisperformance.com/covid19> - Physical exercise (for 6 weeks) has been useful to help return to a normal life - Headspace and creativity important to manage stress and fatigue.  Seems to help with the need to be productive, as well as expressing emotions. - Pain management therapy - Speaking with family and keeping going whilst thinking positive.   **Anything that may be difficult to do online? potential problems?**   - Worksite visits - when OT needs to go and see the workplace (heavy moving and handling jobs to understand the work context and the person’s job. - Online is better and people can be more honest but at the same time, human touch is important and it could be useful to have a face-to-face follow up. - ‘Filling in forms online does not capture the real you’. Caution (!) need to check in with the patient and ensure that their needs are understood.   **Any adjustment that could be made?**   - Greater time spent on initial assessment - There are very different kind of work and jobs, more intellectual jobs may require much longer due to longer rehabilitation of cognitive problems. The first meeting (assessment) is crucial to understand the situation and work through things individually. It is not just the physical aspect of the job but also the emotional and social responsibility and psychological demand that are important to be considered/covered. - More flexibility in the programme - It is hard to know what problems the person will encounter in the workplace before they return to work. New problems might occur then. - Monitoring and review of symptoms following the return. - Getting help feels very bitty and uncoordinated. the OT should act as a case manager to ensure the participant is getting all the support required. - Liaison with GPs is very important – they are good at using a Fit Note to sign people off sick but not so good at writing a Fit Note to support RTW. - OTs could use the AHP Fit for work report to help the GP understand things better as well giving a copy to the participant and employer.   **Contextual considerations**   - New policies and procedures needed for Long COVID given the wide array of symptoms (mental, physical etc.), making tasks like using a computer tough. | | |
| Workshop 2 (July)  People with lived experience of LC (n=5) and clinicans (n=2). | - Reflect on initial participant and OTs feedback. - Review preliminary logic model. - Review first sessions of VR for COVID-19, reflecting on what worked well and what did not. - Discussion of unanticipated issues and solving problems from stakeholder and OT perspective. - Presentation of initial survey results and interviews from study 1. - Reflection on other research relevant to VR from other research teams, experience of returning to work with COVID-19 from other sources. - What are the pertinent issues from this additional information? - What adjustments to VR need to be made? - Review Initial design of manual for digital VR for persistent COVID-19 symptoms. Discussion of format and content. | | **OT Reflection on the first patient**   - The VR intervention was atypical VR as the participant had already returned to work. – The VR focus was on fine tuning skills at work and finding a way of coping with work as opposed to a graded return to work (as envisaged). - It was sometimes difficult to get ‘other’ people involved (mostly the participant’s partner). Their participation was key given the OT’s concerns that she might be pushing the participant to return to work too quickly as a result of poor understanding of their symptoms. This would have been the opportunity to get more support and some feedback at home. - An hour was sometimes not enough; after 12-weeks, participants were just getting started – more sessions would have been beneficial. - The intervention helped the participant not to return to work too quickly and was valuable to educate the employer. - Involvement from a psychologist would have been helpful. - There is a need to learn to manage the new life with Long COVID (not just work), which takes time and goes against attempting to get patients to return to work as soon as possible. It is salient to consider the meaning of having Long COVID and how it affects direct contacts. - The OT should teach different aspects of life to support the patient to adjust to their new self, which is no lesser/better than pre-covid self, just different. This is a slow process and although return to work is the overarching aim, this requires interim, realistic, smart goals. - 12 weeks is insufficient recommend a longer duration for the model going forward.   **Interview with the participant.**   - The participant commented that the intervention supported them in three different ways: - helped with symptoms of Long COVID as the OT provided exercises to help with symptoms and made them reflect on how their lifestyle was unhelpful (for example, not having a regular sleep pattern. The participant reported that he trusted the OT immediately because she had a lot of experience. - The participant had never been on long term sickness absence and did not know what to do and how. The intervention helped them get in touch with Human Resources (HR) and educate HR on their situation and negotiate return to work. When the participant was too keen, the OT was there to help them reflect on whether they could do what they suggested. They could. This was helpful to not return to work too quickly. Ultimately, the intervention was helpful financially and with the participant’s decision making. - Emotional support was important for the participant. They felt the OT had a lot of empathy for them which was helpful, felt she truly understood his situation. - When discussing what could be improved, the participant mentioned that they were more of a face-to-face person and not keen on digital/remote delivery – although they understood the reasons. The participant thought that human contact would have been helpful despite their strong fear of covid, as they trusted medical and health care staff to be safe. The participant thought that it could be restrictive for some people who are not IT savvy. However, they did not feel that the delivery method affected their relationship with Julie or the intervention. - The participant also thought that they would have benefitted from further sessions, mostly for emotional support. They explained that as they were now on a phased return to work, they were feeling under pressure and were concerned about making the wrong decisions. As such an occasional follow up session would be useful. The participant felt having the OT fighting their corner would give them confidence. At the time of the interview the participant did not know when their hours were due to increase as part of the phased return, and they were not fully confident that they could increase their hours. - Overall, extremely pleased with the intervention and what they had achieved and talked positively of the OT. - **Workshop participants reflected on the proposed intervention and the first participant.** - On the importance of involving partners, however, that some patients may not have partners which makes the progress harder given the reduced amount of emotional support at home - One PPIE representative expressed shame when explaining to their work colleagues and managers that they have Long COVID, due to a lack of understanding, emphasising the need to educate employers. There seem to be a preconception that managers know because they are managers. Given the drastic emotional change, educating the employer is key to the intervention. - Helpful – Cognitive Behavioural Therapy (CBT). Partly related to processing traumatic experience and then also learning to live with symptoms rather than hoping for them to go away. Helps with neuroplasticity related to an anxiety response e.g. heart racing, feeling hot. Yoga – gentle strengthening and learning about how to access and stimulate parasympathetic system. Movement and activity to prevent muscle waste and deconditioning. Recumbent exercise machines could help. Sow exercise e.g. Thai chi. Helps to prevent chronic pain/anxiety etc. Skill-building e.g. CBT and mindfulness “relearning”. - One PPI member had had Long COVID for 18 m and still wondering how long it will go on – it is a long-term condition and so should be treated as such. Relates to workplace policies, disability definitions etc. - Interventions should address home routines and structures as well as work ones, stress and fatigue-management. Assessment and intervention by OT should be very holistic – OTs may need to think outside their usual areas of expertise and especially look at respiratory medicine. - Diet changes – healthy eating. Slow energy releasing foods. V important. Zoe app Long COVID video – very good to watch. Some people with comorbidities, low socioeconomic and lack of general knowledge about food may need additional support e.g. referral to dietetics, nutrition, gastroenterology etc. - Women – menstrual cycles might have changed radically e.g. stopped cycles and perimenopausal; GP Louise Newson (national menopause expert) is doing talks for women with Long COVID. - Relapsing has been an issue – doing less than you think you can do seems to help and ensuring work knows about it and that policies, relationships at work permit rapid fluctuation of work duties and absences from work.   **Reflections on Stage 1 interview findings**   - Reasonable adjustments v important; e.g. working 1 day at home, using a lift restricting moving, walking, talking (SOB, inflammation on chest wall), drinking lots of water, damp environment useful, lots of breaks e.g. paid lunch break/other breaks, use of natural breaks e.g. toilet + making drink, workplace accepts the person’s use of mindfulness techniques in the workplace e.g. quiet moments away from colleagues, lying down, resting on the desk etc. - Exercise tolerance much more reduced due to reduced oxygen uptake. Plus, core stability seems to be reduced that affects use of upper limbs and strength. - POTS: standing up from sitting and then maintaining standing – feel dizzy, nauseous and racing heart plus oedema, compression garments use. - Facilitators (what helped/would help them to return to work): - Having a work buddy to assist them could be useful. This person could cover for their work when they are on sick leave and help them catch up on what was missed, thereby relieving pressure in the event of an “off” day and to assist them with more physical tasks. This would allow for a slow/gradual return. - Environmental changes to manage symptoms. E.g. regular/longer breaks and taking breaks in a quiet room. The quiet space was also mentioned for work itself, for example, working in an office where they do not get disturbed to help with difficulties concentrating. - Policy flexibility in the workplace. For example working from home , which allows for greater flexibility. Many nurses were allowed administrative workdays to facilitate this. This also removed travel to work (see below). However, they found that they work longer hours than expected. - Having a supportive manager and team who understand the issues or make the effort to was perceived as helpful, as it brought a more positive atmosphere and less pressure regarding returning to work too soon. It was found that it also made it easier to communicate needs and requirements. - Having regular (weekly) assessments to feedback on performance and review needs to ensure the employer knows what to expect. This would help adapt to improvements but also if a relapse occurs. It also promotes efficient communication.   **Barriers (factors preventing return to work):**   - Long COVID symptoms affect work directly and cause concerns about job performance. - Commute sometimes adds over an hour to the working hours and is only rarely considered by employers in phased returns. This put them at risk, mostly on the way back home. Those using public transports who struggle to get a seat may experience exhaustion before starting work. - Fear of catching COVID-19 again and making things worse. Most people interviewed were nurses and teachers, whose work environments meant infection risk could not be mitigated. - Current policies and procedures around returning to work not reflective of Long COVID, for example the standard phased return does not consider Long COVID symptoms like exhaustion, thus delaying full return. Some phased returns fail to adapt the role-based symptoms. - Lack of understanding and compassion from colleagues, including criticism for not having returned to work, creates a negative and unwelcoming atmosphere and deters RTW.   Workshop 2 also considered emerging evidence from COVID-19 rapid guideline: managing the long-term effects of COVID-19, National Institute for Health and Care Excellence (NICE), Scottish Intercollegiate Guidelines Network (SIGN) and Royal College of General Practitioners (RCGP)^1,2^  **Adjustments needed to Covid-VR intervention**   - Feedback on performance/needs - Dealing with intrusive worries on an ongoing basis - Frequent meaningful communication between parties - Consider the impact of the whole day including travel to work. - Consider healthy eating advice e.g. slow-release energy foods, sleep hygiene, the effect of menstrual and menopause. - Include discussion re relapse, regular monitoring and review - Explore workplace policies for Covid-relevance. - Explore opportunities for flexible working and reasonable adjustments. - Explore options for work buddies. - Education for employers - Consider referrals for psychological support and unexplained, possible Covid-related symptoms e.g. POTS - Include links to resources for participants shared by PPIE members e.g. the online/virtual ENO Breathe programme, compiled by UCL and Arts Council England | | |
| Workshop 3 (August)  People with lived experience of LC (n=3) and clinicians (n=2). | - Reminder of study aims. - Review Intervention design based on the 6 participants’ intervention content and dose data (how much, how often, when and where?). - Present qualitative interview ‘acceptability and usefulness’ findings based on participants experience of intervention. - Feedback from the OTs on experience of delivery - Review of actual intervention against proposed NHS E Covid-19, tiered model to determine what is Covid Specialist VR? - Discuss intervention changes and recommendations for clinical practice | | **Workshop participants reflections.**   - Psychological issues are long term. Opportunities to (re)access psychological support or access this later. - Normalising that physical illness has psychological impacts- patients may not be ready to explore this part of the intervention, maybe introduce this in later sessions (middle to end of the intervention). Interplay between symptoms and anxiety. - Getting others (employers, co-workers, family) to understand the fluctuations, can be challenging. - Receiving timely intervention to promote recovery, VS making decisions about return to work.   **Things to consider:**   - Introduce a visit to the Intensive care Unit (ICU) for critical illness- Covid-19 patients. - Inviting family to attend sessions as they witness a lot and could explain situations from an outsider perspective. Family joining sessions can be very important for them to understand fluctuations and impact but also provide consistent support. - Coordination with other professionals is important as it is not only about returning to work but also accepting the situation and dealing with the impact on mental health, and family and friends (who may not understand and not support adequately). - Consider in person face-to-face sessions or have a mix, maybe starting with remote to manage fatigue and then face-to-face to help participant get out of the house – as a good first step for returning to work.   **Is this a specialist intervention?**   - OT (CW) felt that all her participants required specialist help and that specialist help should not be defined by length of sick leave. Her clinical service defined people off work for more than 6/12 as needing specialist help but some Covid patients who were off work for less than this also needed specialist help. On discussion of what triggered the need for specialist help/what made the intervention specialist (tier 3); - - CW reflected that her participants had a pattern of going back to work, struggling and then crashing (boom and bust cycle). This was also true of one of JP’s participants who was already at work. - Challenges in applying fatigue management to the individuals work- especially in patients with high demand jobs e.g. teaching or where there was a culture of working long hours/being constantly in-demands. The participants found it particularly difficult to adjust, be flexible. - Dealing with co-workers and line managers where participants felt misunderstood or excluded. - Dealing with participants in a work identity crisis e.g. those in a highly demanding job that they now find difficult. - Managers wanting to support but not knowing what to do or how or feeling that they had done ‘everything they could’. Managers wanting to know how long it would last and in need of education about what they could do to increase the chance of a positive and sustained return to work - Understanding what reasonable adjustments are and how to get them implemented e.g. Access to work (A2W) (waiting list for A2W was 4-6 months). - Participants who needed longer-term and or more nuanced support – from someone who understands job roles and how to tailor support. - OTs need skills and confidence to talk to and negotiate with employers, including by phone or email. - Participants who needed multidisciplinary team (MDT) input for Long COVID symptoms (Postural tachycardia syndrome (POTS), breathlessness, anxiety). The MDT input regarding work needed co-ordinating. - Challenges in clinical reasoning due to lack of pattern recognition for fluctuating symptoms - Use of acceptance and commitment therapy (ACT) - Ability to give advice about diet, smell and taste, and menstrual cycle   **What was beyond the specialist VR OTs expertise/comfort zone?**  CW referred people on to respiratory physiotherapy as not confident in dealing with breathlessness, POTS or participants on beta blockers.  **Revise guiding principles and mechanism to include:**   - **Active listening** - The OT VR can be a validating experience, with the participant being heard and working out the problems. - **Advocacy** - Even articulate and experienced people need support – making line managers understand fluctuating condition is challenging/ causes emotional distress. Vulnerable people can be inadvertently managed out of work without support. HR or OH act on behalf of the company not the employee. A health-based intervention to advocate for the patient/employee is important. - **Timely psychological support** and referrals - **Bespoke**- People have different and often uncertain recovery trajectories. Symptoms fluctuate and may continue in the long term. Ensuring patients accept that symptom management is key to recovery and return to work. - **Employer engagement, educating employers**, colleagues, and others about the impact on the individual and on the work role. - **Family involvement** - Education for/ involvement of family in sessions where appropriate - **Dealing with uncertainty**, managing expectations and acceptance of symptoms, dealing with boom-and-bust participants who overwork, struggle and crash, struggling to accept symptoms. - **Regular review of goals** as patients often overambitious and moderate gaols during the intervention. - **Negotiation** - Complex work politics, including Unsupportive work colleagues and line managers affected the client's ability to get on at work- union involvement in 3/6 cases. OTs require negotiation skills, experience of meeting employers and unions and knowing what to anticipate, how to behave and what to say in those situations. - **Signposting and referral** to other services as needed. Building a resource of psychological support options, breathing and relaxation training and classes, exercise rehabilitation, identifying referral mechanisms for specialist (medical) investigations for red flags. - **Multidisciplinary Team (MDT) working –**    - Participants needed MDT input that required co-ordination, e.g. Physiotherapy for postural tachycardia syndrome (PoTS) POTS and breathlessness; Psychologist for anxiety | | |
|  | | | | | |
| **Supplementary Material 3 - Interview Topic Guide** | | | | | |
|  | | | | | |
| Over the past few months you have been receiving help/advice/support from an occupational therapist in relation to your work/ to help you return to work as part of the Covid-19 VR study. These questions are about the help/advice/support you have received: | | | | | |
| **Questions** | | **Prompts** | | **Theoretical Framework of Acceptability** | **International Classification of Function (ICF) VR Core Sets** |
| Can you start by telling us about the help/advice/support you have received from the OT as part of this study?  What do you think the help/advice/support was trying to achieve? | | **What did the OT do**?   - Assess impact of Covid-19 on you/ your ability to do your job? - Help you plan a return to work/prepare you for RTW? - Help to coordinate your return to work (e.g. by talking to others involved in your recovery/care/ RTW inc. your employer) - Give you/ your family & or employer information/education/ advice? - Provide emotional support? - Negotiate a phased RTw? - Monitor your RTW and Provide feedback on your work performance? - Address issues that arise at work? - Explore alternatives (roles/ responsibilities/ job) where unable to return to existing role/job?   **What advice were you given**? | | **Intervention coherence** | **Activities and Participation**   - **Acquired new skills** - **Handling stress and other psychological demands** - **Complex interpersonal interactions** - **Acquiring, keeping and terminating a job**   **Environmental Factors**   - **Immediate family** - **People in positions of authority** - **Health services, system and policies** - **Labour and employment services, systems and policies** |
| How did you/do you feel about the help/advice/support you received from the OT?  What were the benefits of the intervention from your perspective? | | - Were you happy with the content of the intervention? - Is there anything that you would have liked but did not get? (time, resources, length of the intervention)? | | **Affective attitude**  **and**  **Perceived effectiveness** |  |
| How has it helped? | | Helped in a return to work?  Helped in keeping your job or finding new work ?  Helped in dealing with people? E.g family, employers, occupational health, unions, people in positions of authority  Advised on/ helped to resolve pay issues?  Helped in legal/ policy/ dispute discussions?  **Did the OT**  Consult with employment services/ occupational health?  Negotiate RTW/ phased return with employers?  Negotiate changes to your job, role or responsibilities e.g. changes in working hours, extra breaks, equipment, home working, additional support with tasks (known as job adaptations/ accommodations) that enabled you to do your job?? | | **Intervention coherence**  **And**  **Perceived effectiveness** | **Activities and Participation**   - **Acquired new skills** - **Handling stress and other psychological demands** - **Complex interpersonal interactions** - **Acquiring, keeping and terminating a job**   **Environmental Factors**   - **Immediate family** - **People in positions of authority** - **Health services, system and policies** - **Labour and employment services, systems and policies** |
|  |  |  | |  |  |
| - How did the intervention help in your recovery? | | Did the Covid –19 result in problems (e.g. fatigue, difficulty concentrating, memory problems, physical disabilities) that affected your ability to work?  Did the OT develop programmes to overcome these problems/ assist in your recovery?  Eg improving stamina , concentration, abilities specific to the job or role | |  | Energy and drive functions  Higher cognitive functions  Exercise tolerance |
| How much of an effort is/was it for you to engage with the intervention? help/advice/support you received? | | How easy was the advice/ rehabilitation it to follow?  **Digital intervention:**  What do you think of the delivery methods of the intervention?  Were there any challenges?  Did the remote delivery affect your relationship with the OT/other clinicians?  Generally, how well did the technology work for you?  If experienced any difficulty with technology:  Was there any technology/device(s)/software that would have helped but you didn’t have? | |  |  |
| - Was there any help/advice/support that didn’t feel right to you or that you disagreed with? - Did the help/advice/support have any negative consequences for you? | |  | | **Ethicality** |  |
| - How well do you think the help/advice/support (intervention) is working/has worked for you? | | Have you achieved the goals you set out to achieve?  Have you acquired any new skills? E.g. fatigue management, anxiety management? | | **Perceived effectiveness** |  |
| - What has it cost you in terms of money, time, other resources to engage with the help/advice/support from the OT (and CP if relevant)? | |  | | **Opportunity costs** |  |
| How confident are you that you can act on the help/advice/support from the OT? | |  | | **Self-efficacy** |  |
| - How do you think we could measure readiness for return to work? as there is no existing valid measure for survivors of COVID-19? - Were the questionnaires that we used acceptable? - Did you experience any improvements from attending the class that we didn’t measure? | |  | | **Perceived effectiveness**  **Outcomes/Measures** |  |

**Supplementary material 4: Baseline and outcome measures**

| **Name** | ***Items*** | ***Time to complete (min)*** |
| --- | --- | --- |
| Patient Health Questionnaire (PHQ-9)^[[1]](#endnote-1)3^ | 9 | 2 |
| Generalised Anxiety Disorder Assessment (GAD-7) ^4^ | 7 | 1.5 |
| Trauma Screening Questionnaire (TSQ) ^5^ | 10 | 2 |
| Fear of COVID-19 scale ^6^ | 7 | 1.5 |
| COVID-19 Stigma Scale ^7^ | 6/12 | 1.25 |
| Brief illness Perception Questionnaire (BIPQ) ^8^ | 8 | 2 |
| World health organisation Disability Assessment Schedule (WHODAS-ii) ^9^ | 12 | 5 |
| MRC Breathlessness Scale ^10^ | 1 | 1 |
| Chalder fatigue Scale ^11^ | 11 | 2.15 |
| Work and Social Adjustment Scale (WSAS) ^12^ | 5 | 2 |
| Return to Work Efficacy Scale ^13^ | 19 | 3.5 |
| Financial impact | 8 | 4.5 |
| Work Productivity and Activity Impairment Questionnaire: General health V2.0 ^14^ | 6 | 4 |

**Supplementary material 5: Guiding principles and programme theory**

|  | **Guiding Principles:**  **Key issues identified from qualitative interview data and conceptual testing**  **(Phase 1&2)** | **Design Objectives** | **Key design feature(s) of the intervention** | **Programme Theory**  **Mechanisms (M) and Outcomes (O)** |
| --- | --- | --- | --- | --- |
|  | **LC symptoms** |  |  |  |
| **1** | Patients struggle with physical symptoms (e.g. fatigue, breathlessness, chest pain), which pose main barriers to returning to work. | To identify and address physical problems that affect workability and work/life balance. | Fatigue management and breathing strategies; referrals for intervention e.g. physiotherapy, and or further investigation. | **Identifying Covid/illness Impact**: If OTs assess work capability (M), and identify Long COVID impact on the work role (M), patients understand the impact of Long COVID on workability (O), enabling informed RTW decisions (O), the formulation of RTW/Job retention plans (O) and implementation of work-focussed solutions including strategies to self-manage/address limitations (O) |
| **2** | Cognitive symptoms, affecting mostly memory, decision-making and concentration impact confidence and the ability to work. | To identify and assess the impact of cognitive impairments on workability and job role. | Educate patient about cognitive impairment; explore how this affects patient and identify acceptable coping strategies | **As above** |
| **3** | Impact of illness and symptoms on psychological wellbeing and confidence and the ability to work | To identify and assess impact of Long COVID on psychological wellbeing and confidence and work ability | Active listening and informal psychological support; activities to build competence and confidence in work related activities; determine the need for referrals; empower patients to self refer for mental health support services e.g. IAPT; liaise with clinical psychology; | **Timely psychological support (M)**. If OTs screen for common mental health problems (M) and **signposts or refers** to CPs for psychological support / interventions (M), then patients will learn to self-manage mental health problems (O), resulting in improved psychological wellbeing (O), reduced sickness absence (O) →more sustainable work post Covid-19 illness (O) |
| **4** | Symptoms variability (from day to day) make it difficult to adhere to a routine | Determine baseline activity levels; identify patterns to determine progress | Activity monitoring; re-establishing roles and routines. Work preparation activities inc.plans for how to cope on not so good days for pt, family and employer. | **Optimising self-efficacy:** If patient is aware of support/ how to access it, and feels empowered to monitor and manage symptoms at work (M), then hope for a successful RTW/Job retention is fostered (M), increasing the likelihood of positive psychological adjustment (O), engagement in work-focussed solutions (O) + increased confidence in ability to work following Covid-19 (O) |
|  | **Employer and colleagues** |  |  |  |
| **5** | **Failure to understand each individual reaction/adaptation to Long COVID is unique.** | Determine employers understanding re Covid and the patient; identify usual RTW procedures and how much flexibility they have | Educating patient to be honest about symptoms and needs; educating employer on the impact of Long COVID; advising on work adjustments and adaptations to job role and responsibilities. | **Employer engagement and education:**  If effective communication with employer is established early in the rehab process (M), and **individually tailored** Long-Covid educational materials are provided (M), employers, understand the impact of Long COVID on individual's workability (O). High employer/OT/CP engagement in solution focused options for RTW (O) + employer more willing to explore modifications/alterations to work role or conditions (O)+ RTW/Job retention plans implemented. |
| **6** | **Standard return to work procedures may not be appropriate to Long COVID** | Identify usual RTW procedures and how much flexibility they have | Workplace meeting with employee, line managers/HR/employer representatives and explore acceptable RTW plan for employee/er with regular reviews; provide Long-Covid educational materials | **Accommodating Illness at work:** If OTs **engage with employers**, Long-Covid educational materials are provided and workplace meetings are held to **explore options** for RTW, with regular review, then employers more willing to implement adaptations to work role/ responsibilities/ or conditions to support RTW/job retention. |
| 7 | **Work colleagues , HR and managers do not understand Long COVID and the related difficulties** | To identify threats to/ enablers of work sustainability | Long term monitoring and review; strategies and rules for coping with symptom variability; empowering patient to educate/offer education for co-workers; provide educational materials; educating employers and work colleagues on the impact of Long COVID. Advocacy support with education and employer engagement. | **Identifying Covid/illness Impact:** If OTs assess patients work capability, and identify Long COVID impact on the work role (M), and **engage and educate employers** (M) patients, employers HR and work colleagues understand the impact of Long COVID on individual's workability (O), threats to sustainable work identified (O), enabling informed RTW decisions (O), the formulation of RTW/Job retention plans (O) and implementation of work-focussed solutions |
|  | **Role at work** |  |  |  |
| **8** | **Patients current role is not compatible with their Long COVID symptoms** | To assess limitations in workability and assess job role and responsibilities; identify work ability | To explore role adaptions and alternative roles within the workplace where RTW in existing role not feasible, provide support in exiting existing job and assist with finding new work/ returning to work; assist in identifying state benefits. | **Identifying Covid/illness Impact**: If OTs assess work capability, and identify Long COVID impact on the work role (M), patients understand the impact of Long COVID on workability (O), enabling informed RTW decisions (O) and exploration of work alternatives (O) **Accommodating Illness at work:** If OTs engage employers in vocational rehabilitation interventions, employers more willing to implement adaptations to work role/ responsibilities/ or conditions to support RTW/job retention (M). |
| **9** | **Patients lack professional technical knowledge e.g. online working resulting from long absence at work.** | To identify the impact of Long COVID on the work role and responsibilities | To identify workplace support/interventions to regain technical knowledge, skills and confidence and keep up to date with any changes | **Identifying Covid/illness Impact:** If OTs assess patients work capability, and identify Long COVID impact on the work role (M), and **engage with employers** (M) patients and employers, understand the impact of Long COVID on individual's workability (O), and limitations resulting from long absence (O), enabling informed RTW decisions (O), formulation of RTW/Job retention plans (O) and implementation of work-focussed solutions e.g. re-training and work place accommodations (O) e.g. supernumerary support |
|  | **Financial difficulties** |  |  |  |
| 10 | **Financial implications of long-term absence from work and phased return** | Explore financial implications of long-Covid on workability | Signpost to assessment of eligibility for benefits and assist with completing applications | **Identifying Covid/illness Impact**: If OTs assess work capability (M), and financial implications of long-term work absence (M) and phased return, **signposting for assessment** of eligibility for benefits (M), patient is aware of support and financial options (O) options for RTW can be explored (O) enabling informed RTW decisions (O), the formulation of RTW/Job retention plans (O) including phased RTW. |
|  | **Guiding Principles:**  **Issues identified from Feasibility testing**  **(Phase 3)** | **Design Objectives** | **Key design feature(s) of the intervention** | **Mechanisms (M) and Outcomes (O)** |
|  | **Unnerving symptoms e.g POTS increase anxiety** | Ensure symptoms have been investigated medically, where not investigated advise/support referral for further investigation | Offer reassurance, signposting and/or refer to other medical investigation (for red flags), or rehabilitation services as needed, e.g. breathing and relaxation training classes, exercise rehabilitation. Work as part of/liaise with MDT e.g. Long COVID Clinic and or GP where possible. | **MDT Working.** If we implement a ‘VR pathway’ for Long COVID, and employment is discussed as an expected outcome by the MDT (C) + the rehabilitation team has a positive perception that the individual can participate in employment following Long COVID (M) then + high MDT/team engagement in solution focussed options for RTW (O) and RTW is recognized as a health outcome by MDT/ICU/Covid teams (O) →patients expectations managed (O) +increased confidence in ability to work following Long COVID (O). **Case Coordination (M)** (see below) |
|  | **Processing traumatic experience** | Assess if participants need formal psychological support | Active listening to validate patient experience, arrange visit to ICU for ICU patients, signpost or refer for more specialist psychological support e.g. IAPT or CP | **Timely psychological support:** If OTs screen for mental health problems, and educate patient and employers about the impact of Long COVID on mental health + **signposts or refers for psychological support** as needed, then the patient will learn to self-manage mental health problems (O), resulting in improved psychological wellbeing (O), reduced sickness absence (O) →more sustainable work post illness (O) + patient and employer understand the impact of mental health issues on work (O). |
|  | **Long COVID symptoms affect work performance, lead to performance anxiety and work identity issues (esp in high performing individuals)** | Facilitate participants to identify Long COVID impact on work and implement support strategies | Dealing with uncertainty and managing expectations and acceptance of symptoms, dealing with 'boom and bust' participants who overwork, struggle and crash, struggling to accept symptoms. | **Optimising self-efficacy:**  If the individual is able to express their concerns about their Long COVID symptoms during VR and the patient is ready to engage in vocational rehabilitation that supports symptom management, then patients confidence in ability to manage symptoms at work increases (o), resulting in + high self-efficacy of individual (M) →increased confidence in ability to work following Long COVID (O) + high engagement in solution focused work options (O). |
|  | **Learning to live with symptoms** | Facilitate adjustment to working and living with Long COVID symptoms | Managing expectations (as above) and regular review of goals to reflect change and moderate early overambition in line with abilities | **Setting goals.** If employment is discussed as an expected outcome (C) + patient is ready to engage in vocational rehabilitation (C) + vocational goals are set and integral to the rehabilitation plan (M) → high engagement in solution focused work options(O) + expectations around recovery can be managed (o) + increased confidence in ability to work following Covid illness (O). |
|  | **Coordination with other professionals and family and friends (who may not understand and not support adequately) is important for accepting the situation and dealing with the impact on mental health.** | Help others involved with the patient with Long COVID to understand Long COVID impact and implement coping strategies | Education for/ involvement of family in sessions where appropriate, Case coordination across patient, employer and other HCPs involved | **Case-coordination:** If the OT is trained as a case coordinator (C) and communicates effectively with patient, family, employer, and wider MDT (M), then stakeholders collectively understand the individual's work goals (O), + engage in solution focused work options(O) → occupational bond with employer preserved (O) **MDT Working** (as above). |

**Supplementary material 6: Digitally delivered vocational rehabilitation for people returning to work with residual symptoms from COVID-19.**

**Session 1**

Use of digital technology for the intervention – how to use it, data security, what to do if there is a break in internet use or other problems.

Assessment – health, occupation, relationship with employer, other demands and interests before COVID-19. Job situation through the pandemic and relationship with employer. History of COVID -19 – symptoms, tests and management of the condition. Course of COVID-19 remaining symptoms. Impact of remaining COVID-19 symptoms on function and specifically on tasks likely to be impaired in current occupation, including any travel to work as well as work itself and any issues related to Stigma of having COVID-19. Walk through task – a day and then a week of doing usual occupation – what are the barriers and what are the mitigating factors that might help then do their usual occupation.

Homework task: on own or with carer think through symptoms and how they might impact work. Can they think of measures to lessen the impact and make the job manageable.

**Session 2**

Review last session and homework task. Review course of COVID-19 symptoms and impact on function.

Discuss concept of reasonable adjustments under existing law. Discuss likely course of current COVID-19 symptoms - knowledge of the patient, family and employer.

Discuss strategies to lesson impact of key symptoms such as pacing or graded increases in activity for physical fatigue of brain fog, memory aids, coping with anxiety attacks and low mood, tackling breathless. Requires more detailed history of their nature and pattern.

Devise initial plan to deal with one or two challenging features of COVID-19

Homework task. Practice coping strategies set out in session.

**Session 3**

Review last session and homework task. Review course of COVID-19 symptoms and impact on function.

Discuss further strategies if required to address further COVID-19 symptoms and mitigate their impact on function.

Review work situation in relation to other responsibilities. Consider possible return to work or alternatives to current work. Consider how and when return to work should be discussed with employer. Fears and concerns about contacting employer and return to work.

Homework task. Practice coping strategies set out in session.

**Session 4**

Review last session and homework task. Review course of COVID-19 symptoms and impact on function and work.

Refining coping strategies more specifically to work tasks for work preparation e.g. establishing routines with gradually increasing activity; opportunity to practice work skills e.g. computers to increase concentration, walking to increase stamina.

Establishing plan for contacting employer and employment advisors. Identifying reasonable adjustments that might need to be made, likely period of phased return to work and planned route to negotiation with employer. Use of advocates and representatives e.g. union, occupational health assessment.

Homework tasks: practice coping strategies for work preparation. Set up contact with employer.

**Session 5**

Review last session and homework task. Review course of COVID-19 symptoms and impact on function and work.

Review of contact with employer. Any unanticipated changes to job situation. Attitude of employer to return to work, graduated return, temporary or more medium/long-term reasonable adjustments that might need to be made.

Further work preparation tasks.

Need for further negotiation with employer, possibly directly by therapist.

Refinement of reasonable adjustments and return to work plan.

Homework tasks: practice coping strategies for work preparation. Return to work.

**Session 6**

Review return to work. Barriers and mitigating factors. Are reasonable adjustments recognised? Attitude of employer. Review of symptoms, last session and homework.

Is current return to work sustainable or are further adjustments needed. Review for need for additional coping strategies. Tackling stigma. Maintaining work-life balance in relation to continuing COVID-19 symptoms.

Summary of sessions, return to work and discharge.

**Uncomplicated cases will take a minimum of 6 sessions. More complex cases may take up to 12 sessions with more sessional time spent on matters dealt with in sessions 2-6**

**Supplementary Material 7: Tidier Description of the Return to Work after Long COVID VR Intervention**

|  | **TiDier Description** |
| --- | --- |
| **Brief Name** (Provide the name or a phrase that describes the intervention.) | Return to work after Long COVID |
| **WHY** Describe any rationale, theory, or goal of the elements essential to the intervention. | **Goal**   - Return to work after Long COVID is a remotely delivered individually tailored intervention seeking to lessen the impact of COVID-19 by assessing the patient’s role as a worker and finding acceptable strategies to overcome problems directly impacting work activities (e.g., physical, cognitive, psychological, or task/environment-based interventions). - It is a job/education retention intervention involving an occupational therapist (OT) acting as both a rehabilitation provider and a case manager for up to 12-weeks, depending on individual need.   **Underpinning Theory**  Intervention is underpinned by the International Classification of Function (ICF)(World Health Organisation, 2001), a biopsychosocial framework that considers the overall context of the ‘patient’/person. The ICF considers interactions between environmental (e.g., workplace) and covid-related factors, recognising that work disability is created or removed as a result of the interaction between biological, psychological, and social factors (World Health Organisation, 2001). The intervention focuses on modifying work tasks and removing environmental barriers in addition to restorative approaches to promoting functional recovery and psychological adjustment. It also draws on the ‘Work Disability Arena’ or Sherbrooke model (Pransky et al., 2011) which considers the different systems (personal, workplace and healthcare) that surround the worker, and influence return-to-work. Hence, the intervention adopts a case-coordinated approach to cross discipline, cross system, and cross sector communication; the Long COVID OT assumes the role of case manager. |
| **WHAT**  **Materials**:  **Procedures**: | **Materials:**  OTs were provided with a study protocol (rationale and objectives) and materials describing the purpose and rationale for the intervention and its proposed structure, duration, and possible content. As both OTs were experienced neurological vocational rehabilitation specialists, they drew on apriori experience and resources.  Laptop/PC and internet connection and secure web-based meeting environment suitable for remote rehabilitation delivery e.g. MS Teams, Cisco Webex.  **Procedures:**  The OTs arranged up to 12, virtual/online or telephone meetings according to participants preference/need within the 12-week time frame.  ***Delivery of the intervention***  Return to work after Long COVID involved an OT working in a case coordinator role with a wider team of healthcare professionals, employers, family members and/or other relevant parties:   - Assessing the impact of COVID-19 on the patient and their job - Educating and supporting patients, employers and families about the COVID-19 impact on work. - Finding strategies to lessen impact e.g. memory aids, pacing to manage fatigue - Work preparation: establishing routines with gradually increasing activity; opportunity to practice work skills e.g. computers to increase concentration, walking to increase stamina - Liaison with employers & employment advisors to negotiate, plan and monitor a phased return to work.   At initial assessment, approximately three participant generated goals were set and recorded using the Goal Attainment Scaling (GAS) Scale (Turner Stokes, 2006). Participants goals were converted to SMART goals and used as the basis of the intervention and reviewed at the end of the intervention.  **Resources**  Workplace accommodations from the Job Accommodations Network (JAN) <https://askjan.org/>  Goal Attainment Scaling (GAS) <https://www.kcl.ac.uk/cicelysaunders/resources/tools/gas>  Draft Statutory Sick Pay regulations (Coronavirus Amendment), [The Draft Statutory Sick Pay (General) (Coronavirus Amendment) Regulations 2022 (SI 2022/****) - GOV.UK (www.gov.uk)](https://www.gov.uk/government/publications/the-draft-statutory-sick-pay-general-coronavirus-amendment-regulations-2022-si-2022/the-draft-statutory-sick-pay-general-coronavirus-amendment-regulations-2022-si-2022), (accessed 12.5.22 and 5.3.24).  Unison, NHS Covid-19 workforce guidance (England) –a guide for UNISON branches, accessible via [NHS-Branch-guide-England-Covid-terms-and-conditions-and-workforce-policies-1.pdf (unison.org.uk)](https://www.unison.org.uk/content/uploads/2020/06/NHS-Branch-guide-England-Covid-terms-and-conditions-and-workforce-policies-1.pdf), (accessed 5.3.24)  [Allied Health Professions Fitness For Work Report - RCOT](https://www.rcot.co.uk/practice-resources/standards-and-ethics/ahp-health-and-work-report)(Allied Health Professions Federation, 2019)  Work Ability Support Scale Work Questionnaire(Fadyl et al., 2015) accessible via <https://www.kcl.ac.uk/nmpc/assets/rehab/tools-wss-work-questionnaire.pdf>, accessed 2021 and 18.8.25  Business Disability Forum,  <https://businessdisabilityforum.org.uk/knowledge-hub/resource/category/covid-19/> (accessed 19.10.20)  [Fatigue Education Course \| University Hospitals of Derby and Burton NHS (uhdb.nhs.uk)](https://www.uhdb.nhs.uk/fatigue-education-course/) <https://www.uhdb.nhs.uk/fatigue-education-course/>, accessed 05.03.24  [FutureNHS Collaboration Platform](https://future.nhs.uk/system/login?nextURL=%2Fconnect%2Eti%2FL%5FC%5FN%2Fgrouphome), accessible via <https://www.england.nhs.uk/futurenhs-platform/>, accessed 19.08.25  Fatigue  Stripy Lightbulb CIC offers e-learning about Myalgic Encephalomyelitis/Chronic Fatigue Syndrome (M.E./C.F.S.) to professionals (healthcare, education, employers) who have a duty of care over M.E./C.F.S. patients. [M.E./C.F.S. Training for Professionals - Stripy Lightbulb CIC](https://www.stripylightbulb.com/), (accessed x date and 5.3.24)  [Long COVID - elearning for healthcare (e-lfh.org.uk)](https://www.e-lfh.org.uk/programmes/long-covid/), accessible as a registered user of NHS England’s elearning for healthcare website, (accessed 5.3.24)  Future NHS - Living with Long COVID session, accessible via; [Long COVID / Living With session with Care City (youtube.com)](https://www.youtube.com/watch?v=2GaRWbFm3BA), accessed 18.10.21 and 5.3.24  [Researching Long COVID: a new global health challenge - NIHR Evidence](https://evidence.nihr.ac.uk/themedreview/researching-long-covid-addressing-a-new-global-health-challenge/), accessed 5.3.24  **Resources for Patients**  Royal College of Occupational Therapists, How to manage post viral fatigue after Covid-19; Practical advice for people treated in hospital, available at https://www.rcot.co.uk/learn-about-occupational-therapy/ot-advice/manage-post-viral-fatigue-covid (accessed 2021 and 5.03.24).  NHS, [Your Covid Recovery - Supporting Recovery for Long COVID](https://www.yourcovidrecovery.nhs.uk/); Available at <http://www.yourcovidrevovery.nhs.uk>, (accessed 2021 and 05.04.24)  **Breathing**  [STASIS breathing programmes for health](%20STASIS%20breathing%20programmes%20for%20health%20%20(stasisperformance.com)) available at https://www.stasis.life/programs accessed 18.05.21 (recommended by PPI group members) and 19.08.25  **Smell retaining**  [AbScent – AbScent: helping people with smell loss](https://abscent.org.uk/) (accessed 5.3.24)  **References**  Allied Health Professions Federation. (2019, July 30). *The AHP Health and Work Report \| RCOT*. https://www.rcot.co.uk/explore-resources/standards-guidelines/ahp-health-work-report  Fadyl, J. K., McPherson, K. M., Schlüter, P. J., & Turner-Stokes, L. (2015). Development of a new tool to evaluate work support needs and guide vocational rehabilitation: The Work-ability Support Scale (WSS). *Disability and Rehabilitation*, *37*(3), 247–258. https://doi.org/10.3109/09638288.2014.914586;SUBPAGE:STRING:ACCESS  Pransky, G. S., Loisel, P., & Anema, J. R. (2011). Work disability prevention research: current and future prospects. *J Occup Rehabil*, *21*(3), 287–292. https://doi.org/10.1007/s10926-011-9327-z  Turner Stokes, L. (2006). *The North West London Hospitals Goal Attainment Scaling (GAS) in Rehabilitation A practical guide*. https://www.kcl.ac.uk/nmpc/assets/rehab/gas-goal-attainment-scaling-in-rehabilitation-a-practical-guide.pdf  World Health Organisation. (2001). *World Health Organisation. International Classification of Functioning, Disability, and Health: ICF*. https://www.who.int/standards/classifications/international-classification-of-functioning-disability-and-health |
| **WHO PROVIDED** | Intervention provider qualifications  HCPC registered occupational therapists (OT) (BSc OT)  Intervention provider background and experience  Occupational Therapists (OTs) with previous experience of working with people with neurological conditions (essential), Long COVID (desirable) and vocational rehabilitation delivery (essential).  Specific training provided  Due to the expertise and experience of the two OTs, the intervention and trial procedures were explained in a 2-hour virtual meeting with the research team. This comprised an academic OT with extensive vocational rehabilitation experience, and clinical academics with expertise in vocational rehabilitation, long-term conditions, trauma, psychology, psychiatry, and implementation. |
| **HOW** | **Mode of delivery**  Intervention delivered 100% remotely via telerehabilitation (video call or phone). via a University of NHS approved secure online platform (MS Teams or Attend Anywhere) or by telephone (one participant).  **Who attended**  The intervention was delivered 1:1 for 4/6 participants. One participant had a parent present for 9/10 sessions, one had two virtual work meetings which involved their line manager, and a Human Resources manager.  **Other**  Additional time spent in liaison (letters, emails, and phone calls) with the patient, employer/college, family or other healthcare providers and others e.g., Access to Work.  Intervention delivery meetings were frequently re-arranged or cancelled due to clashing appointments or covid-related medical investigations, activities (e.g., work trips/ college sessions), illness (Covid), bereavement, annual leave.  One participant who was working full-time wanted flexible appointments. |
| **WHERE** | **Where provided**  The intervention was typically delivered to participants in their home. However, one video-call session was delivered to a patient sitting in a car (not driving) and one phone call took place with the participant on a bus (both at the participants’ request). One OT was working from home and one delivered the intervention from a work base (NHS Trust).  ***Necessary infrastructure***  *Access to laptops and approved delivery platform for OTs*  *Telephone, Smartphone, laptop or IPad for patients* |
| **WHEN and HOW MUCH** Describe the number of times the intervention was delivered and over what period of time including the number of sessions, their schedule, and their duration, intensity or dose. | **Intervention delivery time**   - Time from consent to 1^st^ session ranged from 1 to 8 days (3,1,8,7,7, 1). - The intervention lasted between 45-85 days (Median 81; IQR  73-82)​ - All six (100%) participants received intervention for the full 12-weeks.  However, one participant had a delayed start and fewer sessions, as they were difficult to contact.   **Number of sessions**   - Range 6-13 sessions per participant (Median 8; IQR 7-10)​.     **Length of sessions**   - Each session lasted approximately 65 minutes (range 15-123) per participant​. - The total face-to-face intervention delivery time (duration) was 3301 minutes (55 hours), range 381-830 minutes (Median 552, IQR 129-443)​. - Non-face to face time total duration (time) was 5220 mins, range from 10-180 (median 780, IQR 563- 1016)​. This included time spent seeking information, writing up a session summary for each participant, liaising with and writing to employers, other health professionals etc., completing allied health and work reports, work reports for the employer and referring to others i.e. Access to work. - In total (face to face and non-face to face), 8521 minutes (142 hrs.) of OT were delivered across the 6 participants.   **Frequency of sessions**  Intervention frequency varied across the 12 weeks​ due to individual circumstances.  The average number of days between sessions per participant ranged from 1 to 14 days (the greatest length between sessions was 17 days (including weekends).  **Summary**  Participants received approximately one session lasting approximately an hour every week for 12 weeks unless personal circumstances e.g. holidays, observing religious ceremonies, death in the family resulted in changes. The OTs spent between 10- and 180-minutes on non face-to-face activities per session. |
| **TAILORING** | Intervention was tailored in content, quantity and duration according to individual need and by mode of delivery due to participant preference.  Both London and Nottingham participants also had to cancel and or re-arrange meetings due to clashing appointments or covid related medical investigations, activities (e.g., work trips/ college sessions), illness (Covid), bereavement, annual leave.  One participant wanted appointments at times to suit her which was difficult as pt working full time 5/7.    The total amount of time (minutes) each Occupational Therapist (OT) spent delivering the intervention is shown in Figure 2. One Occupational Therapist (OT) worked as part of an NHS Covid service with access to/referral mechanisms for other healthcare professional input/ services. The other was independently employed by the study. Both delivered similar intervention components -see Figure 2. Differences were largely due to participant needs and local service availability. For example, in London the OT had access to a clinical psychologist and a rehab assistant with fatigue management expertise, so was able to dedicate more time to these issues.  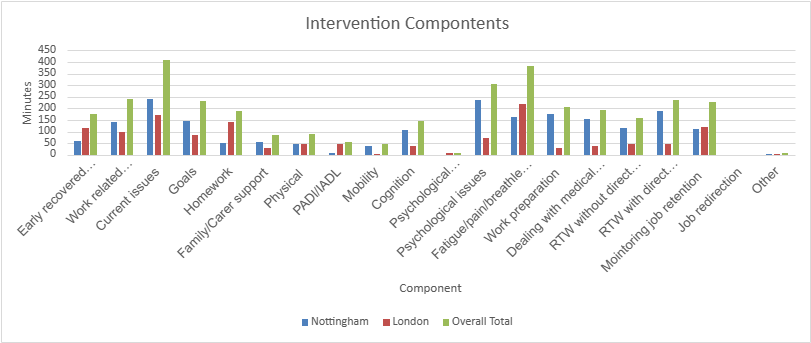  **Figure 2. Time spent on each intervention component.**  During the intervention, some participants had input from other services, some already in place and some signposted or referred by the study OT’s. For example, two participants had help with sleep problems (a sleep apnea clinic, GP + medication), two had physiotherapy (one for a painful shoulder incurred when prone lying in ICU, another for breathing + joint pain), two had input from psychological services, one was referred to a social prescriber.  Following discharge, two participants were on waiting list for OT fatigue management, one was referred to neuro outpatient rehabilitation, another declined a referral to outpatient rehabilitation as they felt they did not have the energy or time to engage in anymore treatment while working. Two participants started exercise for health activities at their local leisure centre after the OT signposted them to this, another was referred to Access to Work (a government scheme to help people with disabilities remain in work) for help with transport to/from work. |
| **MODIFICATIONS** If the intervention was modified during the course of the study, describe the changes (what, why, when, and how). | The proposed intervention was intended to support people to return to work, with a focus on work preparation activities, for example activities to increase stamina and concentration, and planning a phased return and negotiating work accommodations or adaptations with employers. However, as 5/6 participants had already returned to work, it was modified to a job retention intervention, Modifications involved exploring why participants had returned to work, determining current work-related issues, and supporting people with accepting and coping with fatigue and other covid related symptoms, accessing health interventions to support symptom control, ascertain which work modifications would be acceptable and helping participants to communicate their needs to their employer. This was done either by determining which adjustments were needed, reasonable and potentially acceptable to both parties. The OT then either provided a letter or email for the participant to give to the employer (in agreement with the participant) or role-playing conversations in which the participants request modifications from their employer if they preferred to manage this themself. |
| **HOW WELL** Planned: If intervention adherence or fidelity was assessed, describe how and by whom, and if any strategies were used to maintain or improve fidelity, describe them. | As this was a prototype intervention, we planned to measure what was delivered and whether the process was followed but that no formal measures of fidelity, other than content, were recorded. |
| Actual: If intervention adherence or fidelity was assessed, describe the extent to which the intervention was delivered as planned. | **Adherence:**  **The intervention was delivered as planned for** all but one of the prescribed sessions.  This person was still in a ‘boom-and-bust’ cycle, struggling to accept the Long COVID symptoms, and wanted to self-manage the work issues, therefore they declined the OTs suggestions. The OT reported that it took 12 weeks to engage this person and encourage to stop them “fighting” the symptoms.    **Virtual delivery.** It was difficult for the OTs to assess a person’s physical capabilities. For example, the participant who set the goal to walk uphill the shops with friends, had not walked outside unaccompanied. At the time of the assessment, it was difficult to tell if this was due to anxiety, breathlessness or physical ability. It was also difficult for the OT to involve participants’ partners unless the participant specifically wanted them to be involved.  It was easy to arrange meetings with managers/employer representatives as they were used to online meetings and perceived them to be less time consuming than face to face meetings.  Although delivered as planned, notable was that all participants wanted to tell their ‘story’ in great detail (sometimes repeatedly) to feel understood and ensure the OT fully understood what they had been through and the impact it had on them and those around them, before formal intervention could begin. |

**Supplementary material 8. Participants’ goals (GAS)**

| **Participant** | **Participants goal** | | **Smart goal** | **Importance**  **(0,1,2,3)** | **Difficulty**  **(0,1,2,3)** | **Therapist perceived baseline difficulty** | **Achieved** | **By how much** | **GAS Change score** | | | **Variance** |
| --- | --- | --- | --- | --- | --- | --- | --- | --- | --- | --- | --- | --- |
|  |  |  |  |  |  |  |  |  | **Baseline** | **Achieved** | **Change** |  |
| **A** | 1 | To return to work | Identify baseline activity level that participant can consistently achieve, then prepare for RTW using a combination of activities to increase stamina and concentration i.e., walking, computer work and phone calls on Mon, Wed and Friday 1.00 – 3.00 p.m. with 10- 15 min break during this time. | 3 | 2 | -2 | Yes | 2 | 26.7 | 77.1 | 50.4 | Focus of goals changed from returning to work asap to ‘preparing to return’. |
|  | 2 | To remember to do things | Use a notepad or phone to set reminders. Aim to write something 1x a day | 3 | 2 | -1 | Yes | 2 |  |  |  |  |
|  | *3* | To return to work | To feel confident there is a supportive RTW plan in place with employers. Inbuilt review of plan with adjustment to suit needs by week 12.  To clarify the financial situation by week 4. | *3* | *3* | *-2* | *Yes* | *2* |  |  |  |  |
|  |  |  |  |  |  |  |  |  |  |  |  |  |
| **B** | 1 | To be able to go socialize with friends. | To walk to nearest shop to college with a friend and/or have a friend home. | 3 | 3 | -2 | No | 3 | 31.3 | 70.4 | 39.4 | Focus of goals changed during intervention from going out with friends to coping better at college |
|  | 2 | To study for 2-4 hours/day without exhaustion. | To attend agreed set lessons consistently for 2 hours/day. | 3 | 3 | -1 | Yes | 1 |  |  |  |  |
|  | 3 | To feel less tired | To plan and carry out one activity lasting two hours. | 2 | 3 | -1 | Yes | 0 |  |  |  |  |
|  |  |  |  |  |  |  |  |  |  |  |  |  |
| C | 1 | To extend phased RTW whilst managing post-covid symptoms | To maintain a modified work routine, 3 days/week for 6 weeks whilst receiving therapy | 3 | 2 | -1 | Yes | 0 | 37.7 | 50 | 12.3 |  |
|  | 2 | For line manager to better understand condition. | To educate line manager/HR about the long-term effects of Covid and advise on reasonable adjustments to work role. | 3 | 3 | -1 | Yes | 0 |  |  |  |  |
|  |  |  |  |  |  |  |  |  |  |  |  |  |
| D | 1 | To be able to communicate with employer about post covid symptoms and unknown prognosis | To review post covid symptoms and educate patient and employer via letter. To advise on reasonable adjustment until the end of summer term. | 3 | 2 | -2 | Yes | 0 | 30.6 | 50.0 | 19.4 |  |
|  | 2 | To talk about symptoms and their effect at work. | To use two strategies to manage fatigue in the workplace over six weeks. | 2 | 2 | -1 | No | 0 |  |  |  |  |
|  | 3 | To accept condition, self -advocate and self-manage fatigue more consistently at work. | To use two strategies to manage fatigue in the workplace over six weeks. | 2 | 2 | -1 | No | 0 |  |  |  |  |
|  |  |  |  |  |  |  |  |  |  |  |  |  |
| **E** | 1 | Reduce hours or take early retirement | Explore financial implications of reducing hours at work and the criteria for early retirement | 3 | 2 | -2 | No | 0 | 25.5 | 54.30 | 10.6 | Participants’  goals were beyond her control i.e. stop certain duties at work, change others attitudes |
|  | 2 | Stop doing lunch time duty at work | Reduce work duties to what is manageable. | 3 | 2 | -2 | No | 0 |  |  |  |  |
|  | 3 | For work colleagues to understand problems and not to make insensitive remarks | Understand, manage, and explain the symptoms of Long COVID (fatigue, breathlessness, brain fog) and recovery to others. | 3 | 3 | -2 | No | 0 |  |  |  |  |
|  | 4 | To make time for self | Plan a trip with partner. | 3 | 3 | -1 | Yes | 1 |  |  |  |  |
|  |  |  |  |  |  |  |  |  |  |  |  |  |
| **F** |  | To be supported to tell manager that s(he) needs additional support at work | Participant to speak with manager about fatigue and potential adjustments in the workplace. | 2 | 3 | -1 | Yes | 0 | 37.60 | 48.3 | 6.2 |  |
|  |  | To feel like they have better control over fatigue symptoms | Participant to use two strategies to manage fatigue over two weeks. | 3 | 2 | -1 | No | -1 |  |  |  |  |

*Key: Importance and difficulty rated on a 4-point scale, 0 = not at all (important) 0 = not at all (difficult) 1 = a little (important) 1 = a little (difficult) 2 = moderately (important) 2 = moderately (difficult) 3 = very (important) 3 = very (difficult). Goal attainment: Achieved goal (Yes/No): By how much i.e. A lot more achieved = 2, A little more achieved = 1, Achieved as expected = 0. Unchanged = -1, Worse = -2. Therapist perceived difficulty; = -1 unless the patient is as bad as they could be for that particular goal and rated -2 e.g. if the patient has rated the goal as very important and very difficult to achieve*

**Supplementary Table 9. Gas change score**

|  | **Mean score** | |  |
| --- | --- | --- | --- |
|  | **Baseline** | **End of Intervention** | **End-of-Intervention vs. Baseline** |
|  |  |  | **Mean Change (SD, 95% CI)** |
| Goal Attainment Scale^+^ | 31.56 | 58.35 | 26.78 (15.72, 10.28 to 43.28) |
| +: Measures where a higher score represents a positive outcome. | | | |

**Supplementary Material 10: Figure 5: Three Tier Model: Adapted for NHS London’s ‘Vocational Rehabilitation for patients with Post COVID: from ‘Vocational Rehabilitation: BSRM Brief Guidance. Reproduced with permission**
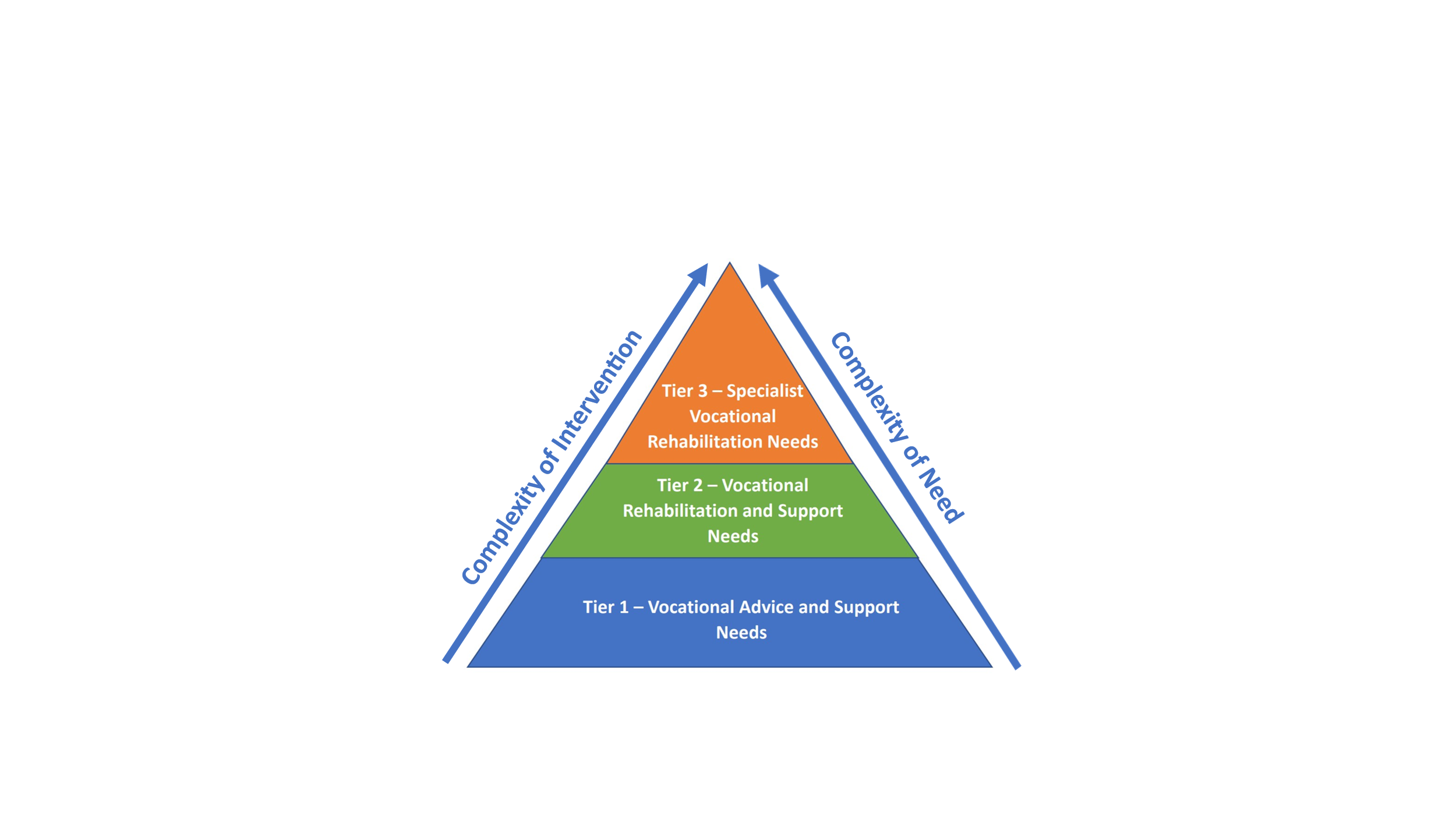


Supplementary Table 11: Pre-covid, pre-intervention and post intervention work status

|  | | **Work Status** | | | | | | | | |
| --- | --- | --- | --- | --- | --- | --- | --- | --- | --- | --- |
|  |  | **Pre-Covid** | | **Pre-Intervention** | | | **Post-intervention** | | |  |
| **Participants** | | **Working Hours/day** | **Working days/week** | **In work** | **Working Hours/day** | **Working days/week** | **In work** | **Working Hours/day** | **Working days/week** | **Change in hours (%)** |
| Site 1 | **A** | 9 | 6 | N | 0 | 0 | GRW | 0 | 0 | N |
|  | **B** | 8 | 5 | Y | 2 | 5 | Y | 4 | 5 | Y (100% ↑) |
|  | **C** | 8 | 5 | Y | 7 | 5 | Y | 7 | 5 | N |
|  |  |  | |  |  |  |  |  |  |  |
| Site 2 | **D** | 8 | 5 | Y | 8 | 4 | Y | 8 | 3.5 | Y (12.5% ↓) |
|  | **E** | 8 | 4 | Y | 4 | 5 | Y | 4 | 5 | N |
|  | **F** | 8 | 5 | Y | 8.5 | 5 | Y | 8 | 5 | Y (6.3% ↓) |

Key: GRW = graded return to work; Y=Yes, N=No

1. References

   1. Shah W, Hillman T, Playford ED, Hishmeh L. Managing the long term effects of covid-19: summary of NICE, SIGN, and RCGP rapid guideline. bmj. 2021;372.

   2. NICE. Managing the long-term effects of COVID-19 (SIGN/NICE/RCGP) | Right Decisions [Internet]. 2021 [cited 2025 Aug 20]. Available from: https://rightdecisions.scot.nhs.uk/managing-the-long-term-effects-of-covid-19-signnicercgp/

   3. Kroenke K, Spitzer RL, Williams JB. The PHQ-9: validity of a brief depression severity measure. Journal of general internal medicine. 2001;16(9):606–13.

   4. Spitzer RL, Kroenke K, Williams JBW, Löwe B. A Brief Measure for Assessing Generalized Anxiety Disorder: The GAD-7. Archives of Internal Medicine. 2006;166(10):1092–7.

   5. Brewin CR, Rose S, Andrews B, Green J, Tata P, McEvedy C, et al. Brief screening instrument for post-traumatic stress disorder. The British Journal of Psychiatry. 2002;181(2):158–62.

   6. Ahorsu DK, Lin CY, Imani V, Saffari M, Griffiths MD, Pakpour AH. The fear of COVID-19 scale: development and initial validation. International journal of mental health and addiction. 2020;1–9.

   7. Do Duy C, Nong VM, Van AN, Thu TD, Do Thu N, Quang TN. COVID-19 related stigma and its association with mental health of health-care workers after quarantined in Vietnam. Psychiatry and Clinical Neurosciences. 2020;

   8. Broadbent E, Petrie KJ, Main J, Weinman J. The brief illness perception questionnaire. Journal of psychosomatic research. 2006;60(6):631–7.

   9. Federici S, Meloni F. WHODAS II: Disability self-evaluation in the ICF conceptual frame. International encyclopedia of rehabilitation. 2010;1–22.

   10. Stenton C. The MRC breathlessness scale. Occupational Medicine. 2008;58(3):226–7.

   11. Chalder T, Berelowitz G, Pawlikowska T, Watts L, Wessely S, Wright D, et al. Development of a fatigue scale. Journal of psychosomatic research. 1993;37(2):147–53.

   12. Mundt JC, Marks IM, Shear MK, Greist JM. The Work and Social Adjustment Scale: a simple measure of impairment in functioning. The British Journal of Psychiatry. 2002;180(5):461–4.

   13. Brouwer S, Franche RL, Hogg-Johnson S, Lee H, Krause N, Shaw WS. Return-to-work self-efficacy: development and validation of a scale in claimants with musculoskeletal disorders. Journal of occupational rehabilitation. 2011;21(2):244–58.

   14. Reilly MC, Zbrozek AS, Dukes EM. The validity and reproducibility of a work productivity and activity impairment instrument. Pharmacoeconomics. 1993;4(5):353–65. [↑](#endnote-ref-1)
