# Supplementary material for: Developing a job retention vocational rehabilitation intervention for people with long covid: a person-based approach
Source: BMJ Open. 2026 May 15;16(5):e109740. doi: 10.1136/bmjopen-2025-109740 (PMC13182320; doi:10.1136/bmjopen-2025-109740)
Supplement: online supplemental file 2 [file bmjopen-16-5-s002.docx]

**Supplementary Material 1:**

**GUIDED^1^ Checklist for ROWTATE Intervention: Complete**

| Item description | Page in manuscript where item is located | Other* |
| --- | --- | --- |
| 1. Report the context for which the intervention was developed. | 4,5,6 |  |
| 1. Report the purpose of the intervention development process. | 7 |  |
| 1. Report the target population for the intervention development process. | 4,5,6 |  |
| 1. Report how any published intervention development approach contributed to the development process | 7,8,9, 10 |  |
| 1. Report how evidence from different sources informed the intervention development process. | Figure 1 and page 8 |  |
| 1. Report how/if published theory informed the intervention development process | 15 |  |
| 1. Report any use of components from an existing intervention in the current intervention development process | 8 |  |
| 1. Report any guiding principles, people or factors that were prioritised when making decisions during the intervention development process. | 8, 13 and Supplementary material 2 and 5 |  |
| 1. Report how stakeholders contributed to the intervention development process | 8, 10, 11, 12, 13 and Supplementary material 2 |  |
| 1. Report how the intervention changed in content and format from the start of the intervention development process | 14, 15, 16, Supplementary material 2 |  |
| 1. Report any changes to interventions required or likely to be required for subgroups. | 34, 35 |  |
| 1. Report important uncertainties at the end of the intervention development process. Intervention development is frequently an iterative process. | 35 |  |
| 1. Follow TIDieR guidance when describing the developed intervention. | 8,18 and Supplementary material 7 |  |
| 1. Report the intervention development process in an open access format. | Submitted to BMJ Open |  |

*e.g. if item is reported elsewhere, then the location of this information can be stated here.
