# Supplementary material for: Developing a job retention vocational rehabilitation intervention for people with long covid: a person-based approach
Source: BMJ Open. 2026 May 15;16(5):e109740. doi: 10.1136/bmjopen-2025-109740 (PMC13182320; doi:10.1136/bmjopen-2025-109740)
Supplement: Supplementary file 3 [file bmjopen-2025-109740.reviewer_comments.pdf]

PEER REVIEW HISTORY

BMJ Open publishes all reviews undertaken for accepted manuscripts. Reviewers are asked to complete a checklist review form and are provided with free text boxes to elaborate on their assessment. These free text comments are reproduced below.

ARTICLE DETAILS

Title (Provisional)

Developing a Job Retention Vocational Rehabilitation Intervention for People with Long COVID: A Person-Based Approach

Authors

Boutry, Clement; Phillips, Julie; Knight, Carina; Holmes, Jain; Patel, Priya; Morriss, Richard; das Nair, Roshan; Douglas, Eleanor; Bolton, Charlotte E; Guo, Boliang; Radford, Kate

---

VERSION 1 - REVIEW

**Reviewer**	**1**
**Name**	**Mcnaughton, Harry**
**Affiliation**	**Medical Research Institute of New Zealand**
**Date**	**27-Nov-2025**
**COI**	**None**

The authors should be congratulated on developing and completing a very difficult project during an extraordinary time. The paper describes the process at considerable length. The 6 participants clearly appreciated the input - close to 25 hours each of OT time. Taken at face value the results regarding work retention and hours of work seem modest, although I accept that the process of managing the participants in their vocational journey proved much more complicated and intense than was initially envisaged. I'm not sure a compelling case for funding of a randomised trial with sufficient power to answer the question of superiority of COVID_VR over a control intervention can be made from these data as suggested in the abstract conclusions.

I thought the Discussion could be more focussed on what COVID-VR offered than the generic prescription from NICE. For me the major strength of the study was the very careful, step-wise construction of an intervention that was most likely to align with the needs of people with Long COVID who wished to return or stay at work.

I found the paper hard to read and not just because of length. There were many grammatical errors which I suspect were due to incorporation of 'tracked changes' without a thorough final proofing. Missing full stops, semicolons following 'and', incorrect tense or verb

agreements were some of these. In the very first sentence of the Introduction, 'caused by' or 'due to' coronavirus 2 is missing. Grammarly or something similar would have identified all of these errors. There was some variety with 'Long COVID' regarding capitalisation and in one case just 'LC'.

At least a couple of the references have inordinately long strings for URLs generated by search engines rather than the home URL itself.

**Reviewer**	**2**
**Name**	**Williams, Parris**
**Affiliation**	**Imperial College London, National Heart and Lung Institute**
**Date**	**09-Dec-2025**
**COI**	**None**

This is an interesting manuscript, and clearly, a large amount of work has been conducted. I have a few minor comments to be addressed.

1- As stated in the manuscript, the number of participants in the intervention group 9 is very low. Were there any further efforts to increase this? As you have previous experience in VR interventions previously did you have a target sample size?

2- It is unclear within your manuscript what happened at phase 3. I understand that participants were recruited, and then did they come to the CRF for screening and baseline questionnaires? Was this conducted over the phone/Zoom? Was formal consent gained? Then, more detail is needed on the intervention. Was it agreed with each participant what the intervention comprised? Did they agree to several sessions? Perhaps another participant flow diagram would be helpful here for a visual representation of the intervention.

VERSION 1 - AUTHOR RESPONSE

The authors should be congratulated on developing and completing a very difficult project during an extraordinary time. The paper describes the process at considerable length. The 6 participants clearly appreciated the input - close to 25 hours each of OT time. Taken at face value the results regarding work retention and hours of work seem modest, although I accept that the process of managing the participants in their vocational journey proved much more complicated and intense than was initially envisaged. I'm not sure a compelling case for funding of a randomised trial with sufficient power to answer the question of superiority of COVID_VR over a control intervention can be made from these data as suggested in the abstract conclusions.

Thank you for this thoughtful comment. We agree that the Phase 3 case series (n=6) was not designed or powered to determine effectiveness or superiority, and that observed changes in work hours/retention should be interpreted cautiously. The primary purpose of Phase 3 was feasibility/acceptability testing and iterative refinement of the Covid-VR intervention within a

Person-Based Approach framework, including understanding delivery dose, content, and contextual/implementation demands (e.g., substantial non–face-to-face coordination time) rather than estimating treatment effects. Consistent with this, we have revised the abstract and manuscript conclusions to avoid implying that these data alone make a compelling case for a definitive, fully powered superiority RCT.

We now state that the intervention appears feasible and acceptable and that the findings identify key uncertainties and design parameters that should be addressed in a staged evaluation (e.g., pilot/feasibility randomised study, implementation evaluation, and outcome selection) before any definitive trial. These revisions clarify the intended contribution of the present study: intervention development and feasibility evidence to inform next-step evaluation rather than efficacy claims.

We also strengthened the limitations to emphasise that work outcomes were not controlled for, participants had variable baseline work status, and the pandemic context likely influenced trajectories and service use.

I thought the Discussion could be more focussed on what COVID-VR offered than the generic prescription from NICE. For me the major strength of the study was the very careful, step-wise construction of an intervention that was most likely to align with the needs of people with Long COVID who wished to return or stay at work.

We agree and have revised the Discussion to foreground what Covid-VR uniquely offers beyond generic guidance. Specifically, we now (i) open the Discussion with a concise summary of the intervention's distinctive "active ingredients" and candidate mechanisms derived from the Person-Based Approach development process and feasibility testing; (ii) explicitly describe how Covid-VR operationalises broad guidance into a deliverable intervention (e.g., structured vocational goal setting and RTW planning, tailored symptom/fatigue management in a work context, employer-facing education/negotiation and coordination, and support for adjustment/identity and emotional restoration); and (iii) reduce NICE and other guideline content to brief context, avoiding reiteration of generic recommendations. These changes sharpen the Discussion's focus on the intervention's design rationale, mechanisms, and implementation implications, consistent with the study's primary contribution: step-wise co-development aligned with pwLC needs.

I found the paper hard to read and not just because of length. There were many grammatical errors which I suspect were due to incorporation of 'tracked changes' without a thorough final proofing. Missing full stops, semicolons following 'and', incorrect tense or verb agreements were some of these. In the very first sentence of the Introduction, 'caused by' or 'due to' coronavirus 2 is missing. Grammarly or something similar would have identified all of these errors. There was some variety with 'Long COVID' regarding capitalisation and in one case just 'LC'.

Thank you for noting these issues. We agree that the manuscript contained avoidable grammatical and punctuation errors, likely introduced during revision and incorporation of tracked changes. We have now completed a full proofread of the entire manuscript and corrected sentence-level issues (punctuation, verb agreement, tense consistency, and inappropriate semicolon use). We also standardised terminology throughout: "Long COVID" is now used consistently, and we removed inconsistent shorthand (e.g., "LC") unless explicitly defined and used sparingly. Finally, we corrected the first sentence of the Introduction to specify that COVID-19 is caused by SARS-CoV-2.

At least a couple of the references have inordinately long strings for URLs generated by search engines rather than the home URL itself.

Thank you for raising this. We identified several references that included inordinately long URLs generated via search engines or containing tracking/redirect parameters. We have revised the reference list to replace these with stable, canonical links, using DOI links for journal articles wherever available and the authoritative organisational/publisher landing pages for reports and books. Specifically, we replaced: (i) the LWW "latest articles/context" URL with the article DOI link; (ii) the Lancet URL containing marketing/tracking parameters (e.g., mkt_tok) with the DOI link; (iii) a Google search URL for the WHO mental health brief with the WHO publication landing page; and (iv) a Google Books URL with the Elsevier canonical book page. These changes ensure all URLs are concise, stable, and journal-appropriate.

Reviewer: 2
Miss Parris Williams, Imperial College London
Comments to the Author:
This is an interesting manuscript, and clearly, a large amount of work has been conducted.
I have a few minor comments to be addressed.
1- As stated in the manuscript, the number of participants in the intervention group 9 is very low. Were there any further efforts to increase this? As you have previous experience in VR interventions previously did you have a target sample size?

We agree that the number of participants receiving Covid-VR in Phase 3 was small. We attempted to maximise recruitment during the study period; however, recruitment and retention were constrained by the timing of the work during the COVID-19 pandemic, service capacity, and the narrow eligibility requirements (e.g., employed individuals experiencing work instability/need for return-to-work support with persistent symptoms). We have clarified these constraints in the manuscript.

Regarding sample size, Phase 3 was designed as a feasibility case series within a Person-Based Approach intervention development programme rather than an effectiveness study; therefore, no formal power calculation was undertaken. We have now made this explicit in the Methods by stating that the planned sample was pragmatic (approximately 6–10 cases) to enable iterative refinement and assessment of feasibility/acceptability. We also strengthened the limitations to

clarify that the sample size and design were intended for feasibility and intervention optimisation and cannot be used to estimate effectiveness or superiority.

2- It is unclear within your manuscript what happened at phase 3. I understand that participants were recruited, and then did they come to the CRF for screening and baseline questionnaires? Was this conducted over the phone/Zoom? Was formal consent gained? Then, more detail is needed on the intervention. Was it agreed with each participant what the intervention comprised? Did they agree to several sessions? Perhaps another participant flow diagram would be helpful here for a visual representation of the intervention.

We agree that Phase 3 procedures were not described with sufficient clarity. We have revised the Methods to include a dedicated "Phase 3 procedures" sub-section that explicitly sets out: identification by the clinical teams; eligibility confirmation and provision of the participant information sheet; formal informed consent (obtained electronically via REDCap); baseline data collection (conducted remotely by the research fellow); referral/hand-off to the OT; delivery format (video/telephone via MS Teams/NHS-approved platform); and the timing of follow-up assessments and the post-intervention acceptability interview. We have also revised Figure 4 to reflect the procedure.

We have also expanded the intervention description to clarify that Covid-VR was individually tailored and agreed collaboratively at the initial OT assessment, including whether employer contact was permitted, session frequency, and the anticipated duration (12 weeks) with flexibility in number of sessions according to need.

VERSION 2 - REVIEW

**Reviewer**	**2**
**Name**	**Williams, Parris**
**Affiliation**	**Imperial College London, National Heart and Lung Institute**
**Date**	**23-Feb-2026**
**COI**	

Thank you for addressing my comments. I believe this manuscript is now ready for publication.
